# Supplementary material for: Genome-wide association study of eosinophilic granulomatosis with polyangiitis reveals genomic loci stratified by ANCA status
Source: Nat Commun. 2019 Nov 12;10:5120. doi: 10.1038/s41467-019-12515-9 (PMC6851141; doi:10.1038/s41467-019-12515-9)
Supplement: Supplementary file 1 — Supplementary Information [file 41467_2019_12515_MOESM1_ESM.pdf]

Supplementary Information for 'Genome-wide association study of eosinophilic granulomatosis with polyangiitis reveals genomic loci stratified by ANCA status'.

Paul A Lyons*, James E Peters*, Federico Alberici*, James Liley*, Richard M.R. Coulson, William Astle, Chiara Baldini, Francesco Bonatti, Maria C Cid, Heather Elding, Giacomo Emmi, Jörg Epplen, Loïc Guillevin, David R. W. Jayne, Tao Jiang, Iva Gunnarsson, Peter Lamprecht, Stephen Leslie, Mark A. Little, Davide Martorana, Frank Moosig, Thomas Neumann, Sophie Ohlsson, Stefanie Quickert, Giuseppe A. Ramirez, Barbara Rewerska, Georg Schett, Renato A. Sinico, Wojciech Szczeklik, Vladimir Tesar, Damjan Vukcevic, The European Vasculitis Genetics Consortium, Benjamin Terrier, Richard A Watts, Augusto Vaglio, Julia U Holle, Chris Wallace, & Kenneth G. C. Smith

Table of Contents

Supplementary Methods.

Supplementary Notes.

Supplementary Figure 1. Manhattan plots showing only directly genotyped SNPs.

Supplementary Figure 2. Enrichment of asthma and eosinophil-associated variants in EGPA.

Supplementary Figure 3. Correlation of estimated effect sizes between primary and replication cohorts.

Supplementary Figure 4. The MHC association with MPO +ve EGPA is localized to the Class II region.

Supplementary Figure 5. Genetic similarity between EGPA subsets and asthma.

Supplementary Figure 6. Contour and density plots of test statistics for quantifying pleiotropy.

Supplementary Figure 7. Genomic features and associations with other traits at non-MHC EGPA-associated loci.

Supplementary Figure 8. Locus zoom plots of loci associated with EGPA.

Supplementary Figure 9. Genetic effects on eosinophil count correlate with risk of EGPA.

Supplementary Figure 10. The *TSLP* promoter region variant rs1837253 has a greater effect size in EGPA than in asthma.

Supplementary Figure 11. QQ plots of genetic associations in EGPA according to increasing degrees of association in IBD.

Supplementary Figure 12. Amino acid positions in HLA-DRB1, HLA-DQA1 and HLA-DQB1 associated with susceptibility to EGPA.

Supplementary Figure 13. Forest plot of Mendelian randomization estimates for the causal effect of eosinophil count on EGPA.

Supplementary Figure 14. Principal components analysis (PCA) of genotype data.

Supplementary Figure 15. QQ plots for all EGPA cases vs controls.

Supplementary Table 1. Criteria for the diagnosis of EGPA from the ‘Study to Investigate Mepolizumab in the Treatment of Eosinophilic Granulomatosis With Polyangiitis’ (MIRRA[$]).

Supplementary Table 2. Breakdown of 542 cases and 6717 controls by country and center.

Supplementary Table 3. Ethics approval from each contributing centre.

Supplementary Table 4. Direction of effect at EGPA variants on eosinophil count and asthma risk.

Supplementary Table 5. Replication cohort case demographics by country of origin.

Supplementary Table 6. Meta-analysis of genetic associations with EGPA in the primary and replication cohorts.

Supplementary Table 7: ANCA status according to country of recruitment.

Supplementary Table 8: associations of ANCA status with clinical features using logistic regression with adjustment for country of origin.

Supplementary Table 9. Meta-analysis of genetic associations with EGPA subsets stratified by ANCA status in the primary and replication cohorts.

Supplementary Table 10**.** Evidence to support biological plausibility of EGPA-associated variants.

Supplementary Table 11: Association of classical MHC alleles with MPO+ve EGPA.

Supplementary Table 12**.** Minor allele frequencies at HLA alleles associated with EGPA stratified by country.

Supplementary References.

Supplementary Note 1: inclusion criteria

There are no validated diagnostic criteria for EGPA. The 2012 Chapel Hill Consensus Conference (CHCC) described EGPA as a disease characterized by 'eosinophil-rich and necrotizing granulomatous inflammation often involving the respiratory tract, and necrotizing vasculitis predominantly affecting small to medium vessels, and associated with asthma and eosinophilia'[1]. However, as acknowledged in the CHCC publication itself, the product of the CHCC is a nomenclature system, and not diagnostic or classification criteria, and thus the CHCC definition is not suitable for diagnosis.

The emphasis on a histopathological definition of EGPA can be traced back to Churg and Strauss's original description of the syndrome, which was made largely on the basis of post-mortem studies of patients with untreated long-standing disease[2]. In modern clinical practice, overt vasculitis is much harder to detect. In a series of 23 EGPA patients published by Reid *et al* in 1998, only 4 met the original histopathological criteria of Churg and Strauss[3]. There are multiple reasons for this. Many patients who present with EGPA are already on chronic corticosteroid therapy for asthma control. Patients who present with organ- or life-threatening disease and the typical clinical, radiological, hematological and serological findings are treated empirically with high-dose corticosteroids, and so if a biopsy is taken it is usually after treatment has been instituted. Affected tissues may not be easily accessible for biopsy, and the tissue samples that are obtained are small, making vasculitis much harder to detect than on post-mortem studies.

Lanham *et al.* first identified the limitations of diagnostic criteria that focus narrowly on fulfilling the pathological features of necrotizing vasculitis, extravascular granulomata and tissue infiltration by eosinophils, since these pathological features often do not co-exist spatially or temporally, or indeed at all[4]. In recognition of the limitations of diagnostic criteria that required histopathological evidence of necrotizing vasculitis and granulomata, Lanham *et al* proposed diagnostic criteria[4] that required the presence of asthma,

peripheral blood eosinophilia (peak count >1.5x10^9/L) and systemic vasculitis involving two or more extra-pulmonary organs. The evidence of vasculitis could be clinical or radiological, and did not have to be confirmed histopathologically. These criteria are not widely used, as they fail to identify the many patients who do not have overt evidence of vasculitis.

The 1990 American College of Rheumatology (ACR) classification criteria were designed to classify patients with already documented vasculitis[5]. The ACR criteria were derived through analysis of 20 EGPA patients and 787 patients with other forms of vasculitis, in which multivariate modelling was used to select a set of 6 features that most effectively discriminated EGPA from other forms of vasculitis when 4 or more were present. In this dataset, the presence of 4 or more of the 6 yielded a sensitivity of 85% and a specificity of 99.7%. However, it is important to note the ACR criteria were not developed for making a diagnosis in individual patients and have not been validated for this purpose[6]. Indeed, The ACR criteria for other vasculitides developed concurrently using this dataset have been shown to perform poorly when used for diagnosis[7]. In the series of 23 patients with a clinical diagnosis of EGPA reported by Reid et al, only 14 met the ACR criteria[3]. Therefore the ACR criteria are unsuitable for most clinical studies.

More appropriate for use in clinical or genetic studies are the recently developed diagnostic criteria used in the Phase III clinical trial “Study to Investigate Mepolizumab in the Treatment of Eosinophilic Granulomatosis With Polyangiitis” (MIRRA: **Supplementary Table 1**)[8]. These define EGPA diagnosis based on the history or presence of *both* asthma and eosinophilia (>1.0 \times 10^9/L and/or > 10% of leukocytes) *plus* at least two additional features of EGPA. Of note, the MIRRA criteria include a wider range of clinical features than the ACR criteria (e.g. cardiac involvement and glomerulonephritis), and also the results of ANCA testing, which was not widely available at the time the ACR criteria were developed.

Supplementary Note 2: comparison of genetic similarity of ANCA negative EGPA and MPO positive EGPA to asthma

We compared *Z*-scores from the MPO+ vs control analysis to *Z*-scores from asthma (which we denote Z_a) using the test statistic $X_{p,}$ defined below. To assess whether $X_{p,}$ was significant, we compared its observed value to distributions estimated under 3 sampling schema. We repeatedly resampled 159 samples with the same geographic distribution as the MPO+ cases, without replacement, under the following three schema:

A) from ANCA- cases, using all controls
B) from all EGPA cases, using all controls
C) from controls, using remaining controls as the control set.

For each draw *i*, we conducted a GWAS case/control analysis using the same methodology and covariates as for the MPO+/control analysis and calculated a value for $X_{p,}$, to generate sets of test statistics X_A, X_B, X_C from draws under schemas A, B and C respectively.

We estimated a p-value for the deviation of X_p from what would be expected under a null hypothesis H_0^{hom} of non-HLA genetic homogeneity between MPO+ and ANCA- subtypes. We assumed a sampling distribution of X_p under H_0^{hom} as

$$X_p|H_0^{hom} \sim N(mean(X_A), var(X_B))$$

Under H_0^{hom}, by assumption, the mean of X_A is equal to the expected value of X_p. The variance of X_A underestimates the sampling variance of X_p, since there are fewer ANCA- cases than EGPA cases in total, so we estimated the sampling variance of X_p with the variance of X_B. We note that we take the sampling variance of X_p to correspond to a sampling schema of ‘select 159 cases from the 534 available EGPA cases’ rather than ‘sample 159 cases

from the EGPA population'. Since X_p is an average across multiple SNPs, we assumed a normal sampling distribution for X_p under CLT.

Using this schema, we obtained a p-value of 0.033 against H_0^{hom}, indicating reasonable evidence for differential genetic basis between MPO+ and ANCA- EGPA outside the MHC region. X_p was typical of the values X_C (quantile 0.46), so we were unable to determine if MPO+ EGPA had any heritability outside the MHC region on the basis of this analysis. Densities of X_A, X_B, and X_C compared to X_p are shown in **Supplementary Figure 5**. When the MHC region was included, MPO+ EGPA was not significantly more or less similar to asthma than ANCA- EGPA (p=0.34).

We show that EGPA and asthma share associated variants (**Table 2**, **Supplementary Table 7**). Therefore the question arises as to whether the variants driving the difference in genetic architecture between ANCA- and MPO+ EGPA are or are not those associated with asthma. Expressed another way, are the two EGPA subsets genetically distinct because one subset more closely resembles asthma, or because they are simply intrinsically different, independent of any relationship with asthma? To address this, we chose a test statistic so as to be maximally sensitive to joint association with asthma and the EGPA subtype (see below), but that retains power to detect different genetic architectures between ANCA- and MPO+ EGPA at non-asthma associated variants. In other words, the value of the test statistic generally reflects 'similarity to asthma' but is also responsive to 'greater overall heritability'.

If the different genetic architectures between ANCA- and MPO+ EGPA were primarily due to different effect sizes at non-asthma associated SNPs, then we would expect that the difference would be largely retained if we removed any dependence between Z scores for asthma and Z scores for the EGPA subtype. To check this, we reproduced test statistics X_A, X_p in the same with the Z_a scores randomly shuffled to give test statistics X_A', X_p'.
This removed any dependence between the sets of Z scores, but retained the marginal distributions of Z scores for asthma and for the EGPA subtypes. We

found that both X_A' and X_p' were indistinguishable from X_C (quantile of mean(X_A') in X_C = 0.95, quantile of mean(X_p') in X_C = 0.94), by contrast to X_A and X_p which were significantly different from X_C. This indicated that the observed difference in genetic architecture between MPO+ and ANCA-EGPA was generally at variants which were also associated with asthma, and that the two subtypes differ in their genetic similarity to asthma.

Graphically, a case in which the EGPA subtype showed no shared association with asthma would appear on **Supplementary Figure 6** as a set of points displaced significantly from the origin along the x-axis, but not the y-axis (variants associated with the EGPA subtype, but not with asthma), and a separate set of points displaced along the y-axis but not the x-axis (variants associated with asthma but not with the EGPA subtype). By contrast, variants associated with both asthma and the EGPA subtype would be displaced from the origin in both the x- and the y-axes simultaneously.

Choice of statistic for pleiotropy

To characterise the degree of pleiotropy between two phenotypes characterised by sets of Z scores, we sought a test statistic to detect concurrently high Z scores in both phenotypes. We considered two metrics for this; for Z-scores $Z_a(i)$ for asthma and $Z_t(i)$ for the trait t under investigation at SNP i of n SNPs in total, these were

$$X_{1,\alpha}(Z_a, Z_t) = \left(\frac{\sum_i |Z_a(i) Z_t(i)|^\alpha}{n}\right)^{\frac{1}{\alpha}}$$

$$X_{2,\alpha}(Z_a, Z_t) = \left(\frac{\sum_i\left(\sqrt{Z_a(i)^4 + 1} + \sqrt{Z_t(i)^4 + 1} - \sqrt{(Z_a(i)^2 - Z_t(i)^2)^2 + 1} - 1\right)^\alpha}{n}\right)^{\frac{1}{\alpha}}$$

with α in {1,2}. Contours of the two test statistics are shown in **Supplementary Figure 5**.

The form of test statistic 2 was chosen so that SNPs with simultaneously high $|Z_a|$ and $|Z_t|$ values would contribute to the statistic, but there would be minimal contribution from SNPs for which the point (Z_a, Z_t) was near the *x* or *y* axis, even if one of Z_a , Z_t were very large.

We determined the statistic to use in the above analysis by determining which statistic best separated values X_B from X_C (defined as in the previous section), assessing separation by the value of a t-statistic score between the two sets of values. The best-performing test statistic was X_1 with α=2.

Concluding remarks

In this analysis, we showed that ANCA-negative EGPA individuals have systematic genetic differences to MPO+ EGPA individuals outside of the MHC region, by establishing that the former showed greater pleiotropy with asthma. The phenotypic similarity of EGPA with asthma more generally indicates that these genetic differences are likely to correspond to clinically important pathophysiological processes. Given that our sampling maintained the geographic distribution of cases, this finding is unlikely to be confounded by geography.

An obvious metric for assessment of pleiotropy between asthma and the EGPA subtype of interest is genetic correlation (r_g). However, estimation of r_g is complicated and estimates have prohibitively large variance when made using the small number of cases in this study. The aim of the analysis described above was simply to indicate genetic differences between the two EGPA subtypes, rather than to estimate genetic correlation. While the metric we used was somewhat simpler, it was difficult to compare its distribution across different study sizes, which necessitated the downsampling of ANCA-negative EGPA cases.

Supplementary Figures

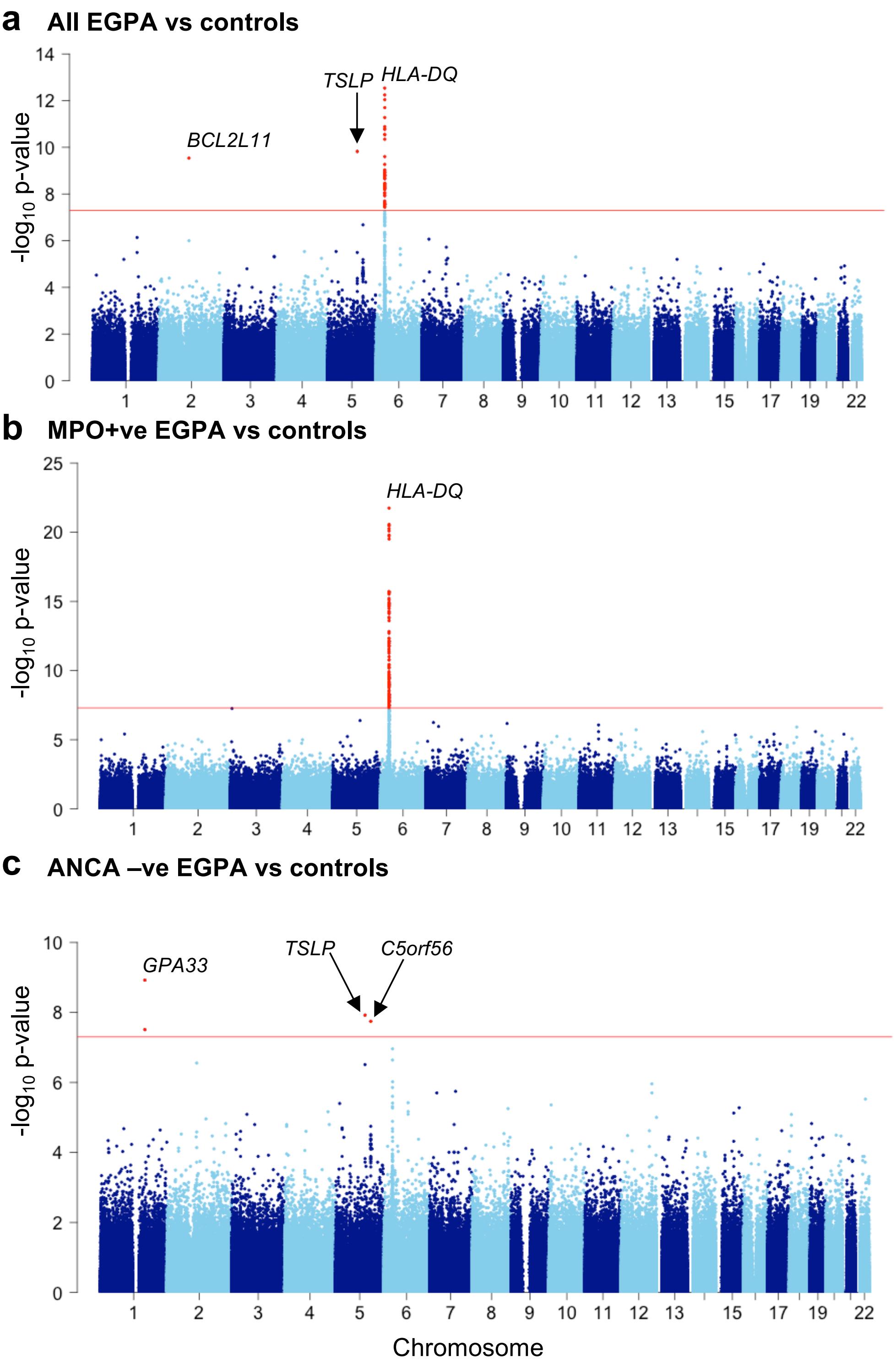

Supplementary Figure 1. Manhattan plots showing only directly genotyped variants. P-values from BOLT-LMM. The horizontal red line indicates genome-wide significance (P $5x10^{-8}$). Significant variants are coloured in red.

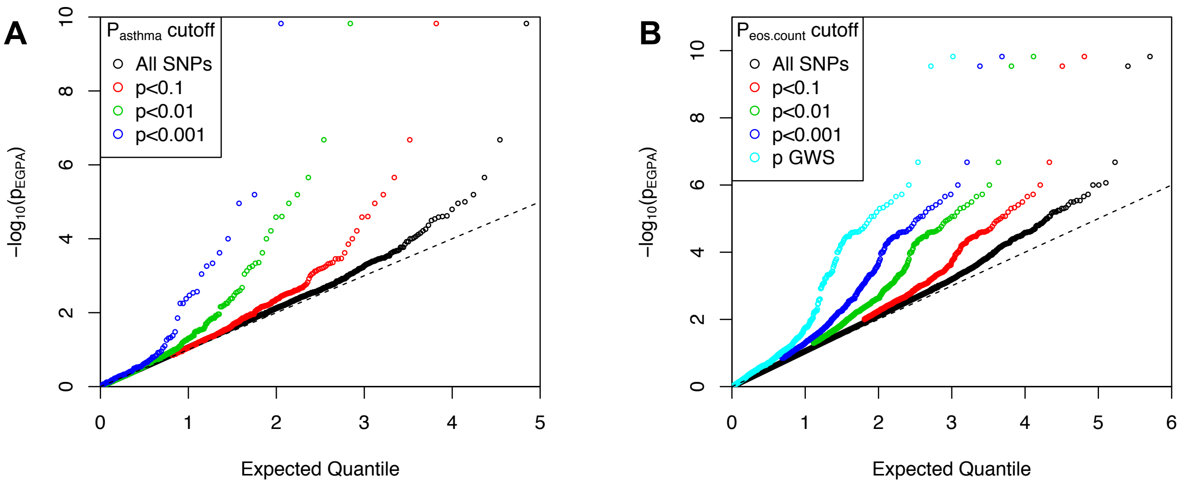

Supplementary Figure 2. Enrichment of asthma and eosinophil-associated variants in EGPA. QQ plots of observed –log10 p-values for the association of genotype with EGPA (from BOLT-LMM) versus the expected –log10 p-values under the null hypothesis of no association, conditional on varying degrees of association with **A**) asthma and **B**) eosinophil count. The MHC region has been excluded. Each circle represents a SNP. The coloured circles indicate sets of SNPs with increasing degrees of association with asthma (panel A) or eosinophil count (panel B). Their QQ-plots demonstrate progressive departure from the line y=x (dotted), indicating shared genetic architecture between EGPA and these traits.

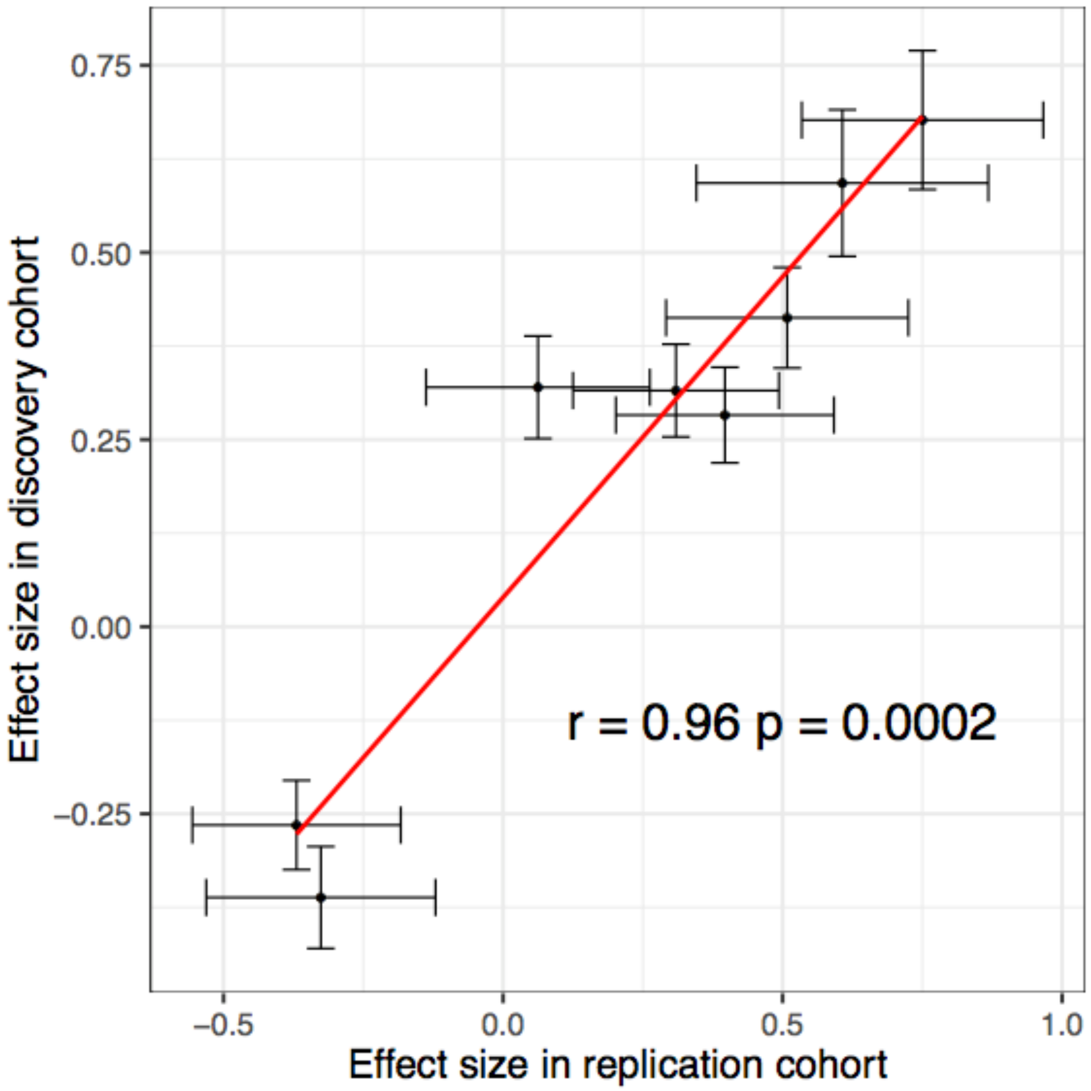

Supplementary Figure 3. Correlation of estimated effect sizes between primary and replication cohorts. Log (estimated ORs) (+/- 95% confidence intervals) are shown. Pearson r = 0.96, p = 0.0002.

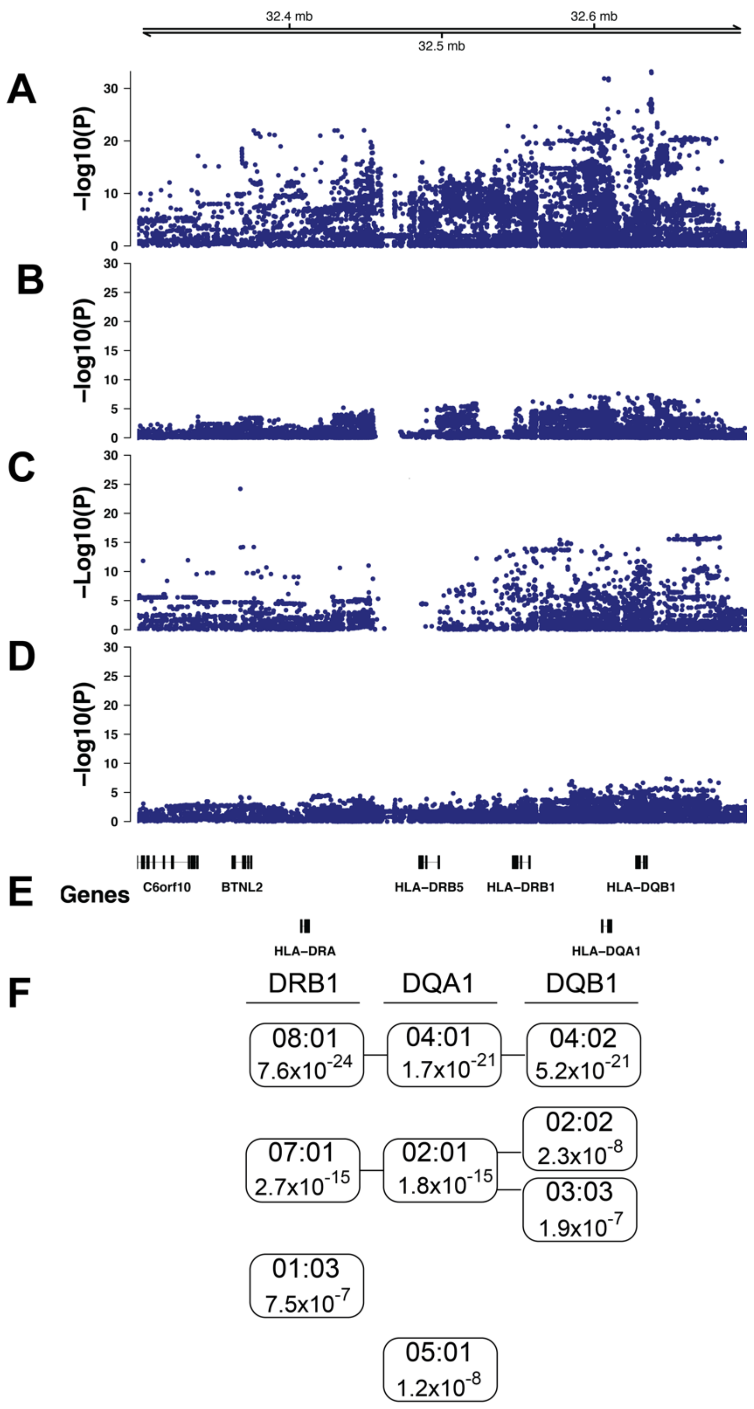

Supplementary Figure 4. The MHC association with MPO+ve EGPA is localized to the Class II region. The MHC association signal in MPO+ EGPA in the primary cohort (A), replication cohort (B), MPO+ ANCA-associated vasculitis (C from Lyons et al [9]) and ANCA –ve EGPA (D). The positions of selected Class I and Class II loci are indicated (E), all co-ordinates are from the hg19 genome build. **(F)** Imputation of classical alleles identifies three MHC haplotypes that confer susceptibility to EGPA.

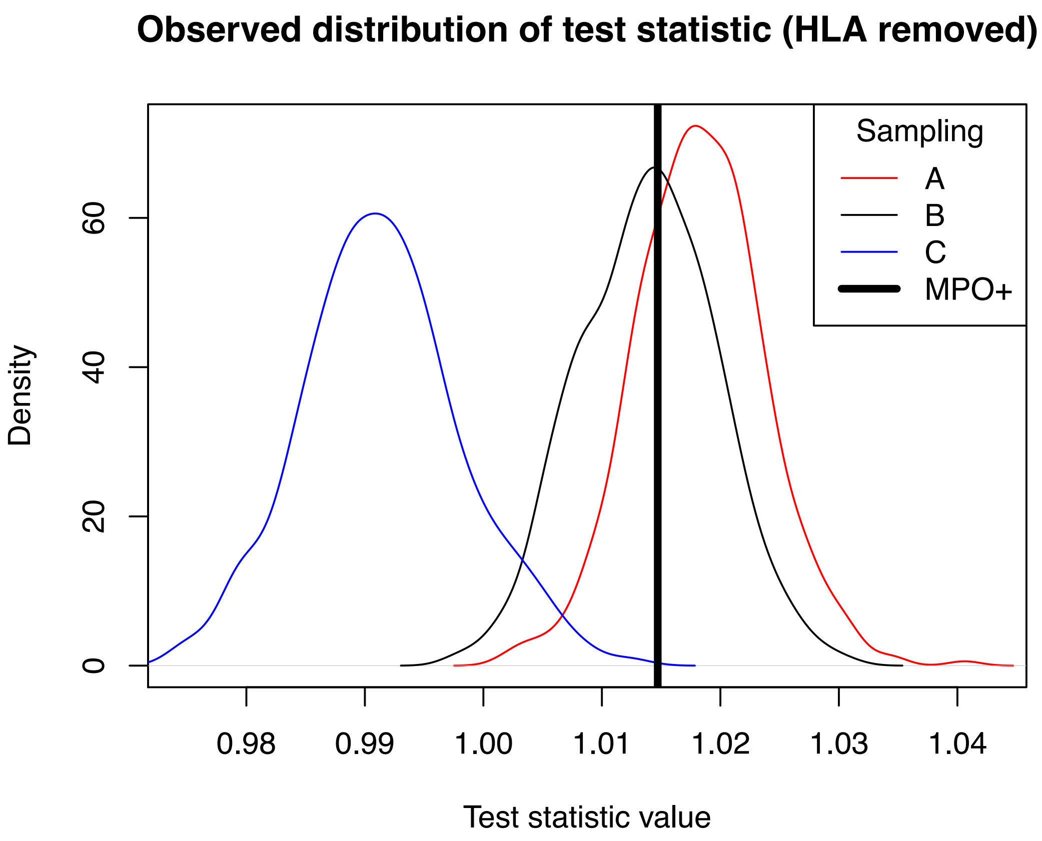

Supplementary Figure 5. Genetic similarity between EGPA subsets and asthma. The distributions of X_A, X_B, and X_C under the sampling schemas described in the Supplementary Note are shown (MHC region removed). Greater right-displacement indicates greater genetic similarity with asthma. Samples from ANCA negative cases (A) show greater genetic similarity with asthma than do MPO+ cases (vertical line) or samples from all EGPA cases (B). MPO+ cases are indistinguishable from controls (C) on the basis of this data.

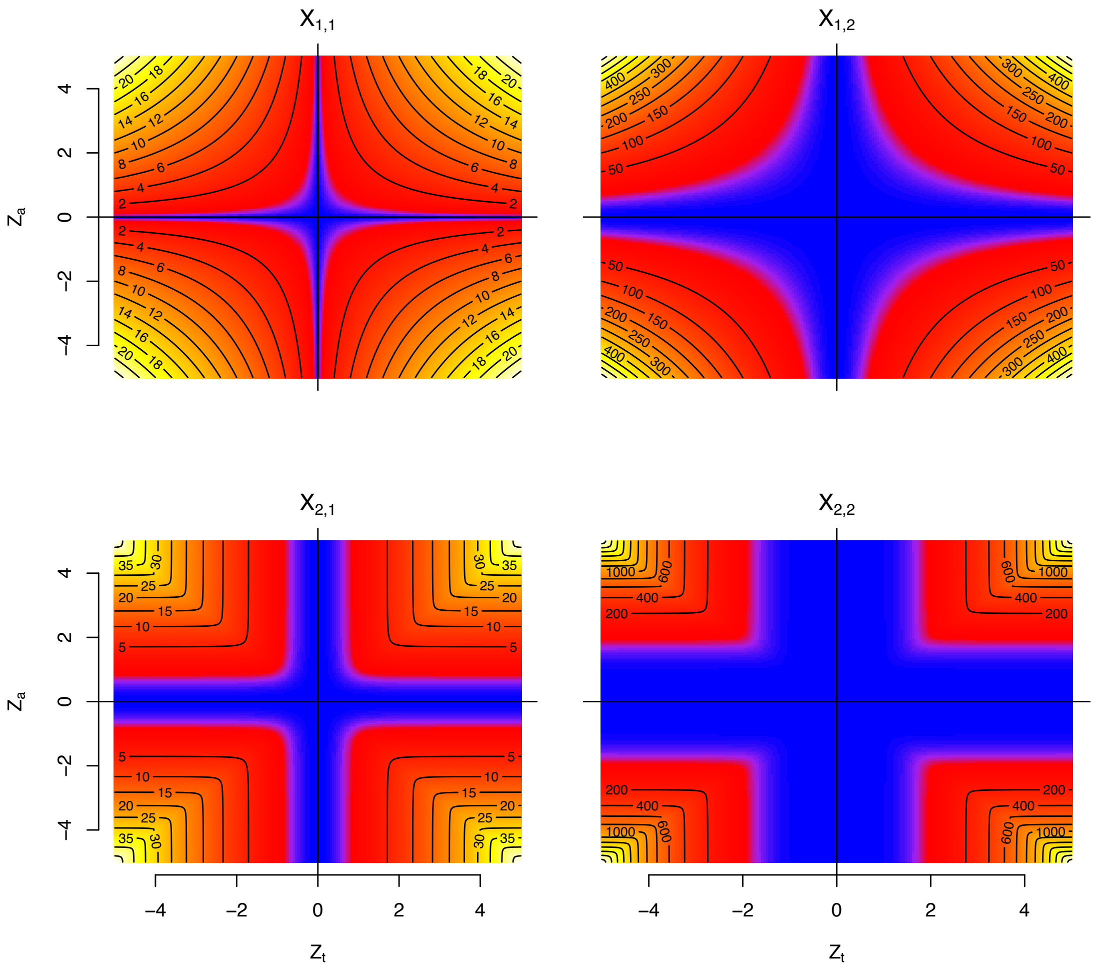

Supplementary Figure 6. **Contour and density plots of test statistics for quantifying pleiotropy.**

Blue colours correspond to values near zero, yellow to large values. The contribution to X_1 of a SNP with (Z_a, Z_t)=(1,x) becomes arbitrarily large as $x \rightarrow \infty$, but the contribution to X_2 is bounded with increasing x.

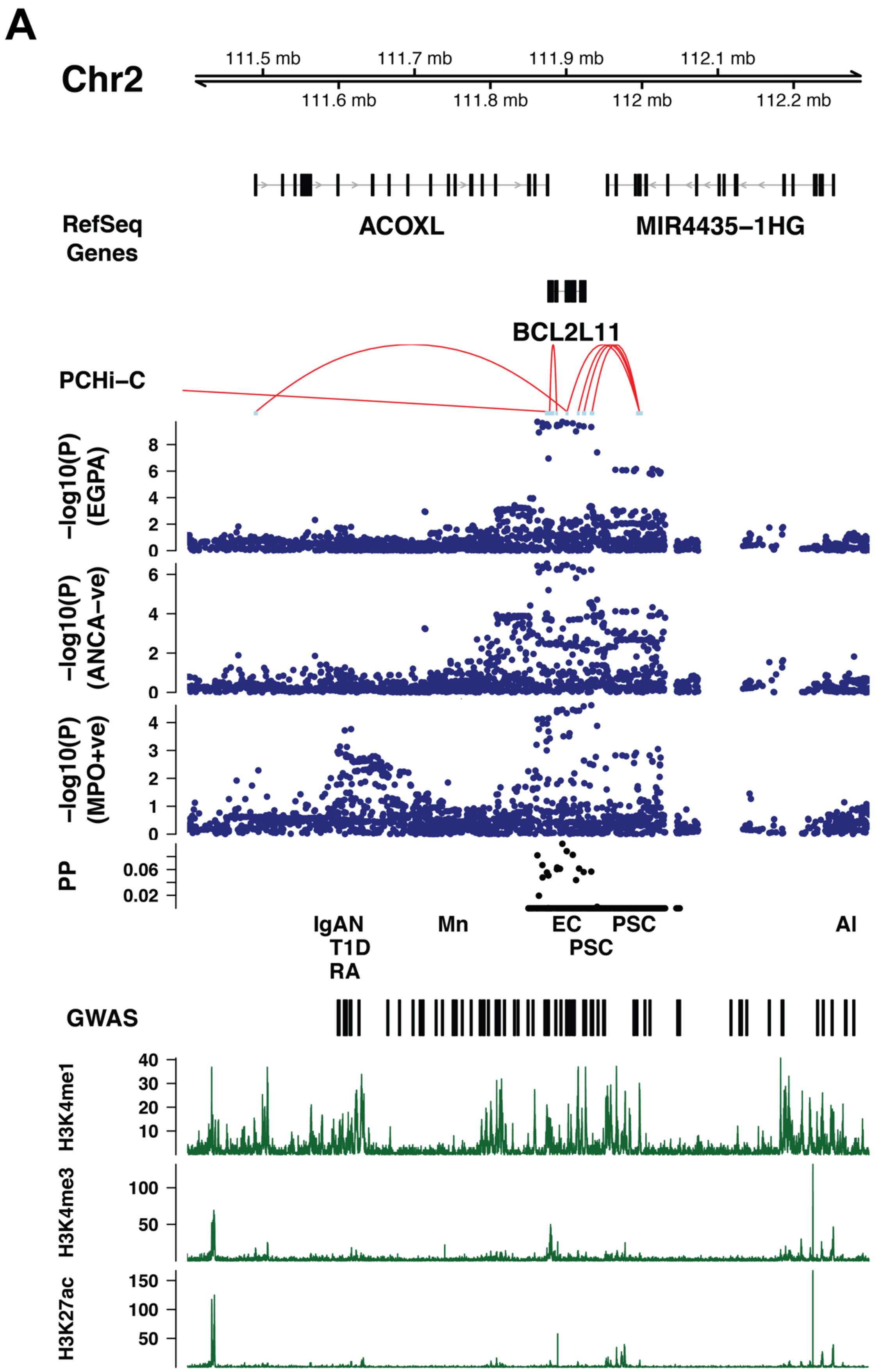
A
Chr2
111.5 mb
111.7 mb
111.9 mb
112.1 mb
111.6 mb
111.8 mb
112 mb
112.2 mb
RefSeq Genes
ACOXL
MIR4435−1HG
BCL2L11
PCHi−C
−log10(P) (EGPA)
−log10(P) (ANCA−ve)
−log10(P) (MPO+ve)
PP
0.06
0.02
IgAN
T1D
RA
Mn
EC
PSC
PSC
AI
GWAS
H3K4me1
H3K4me3
H3K27ac

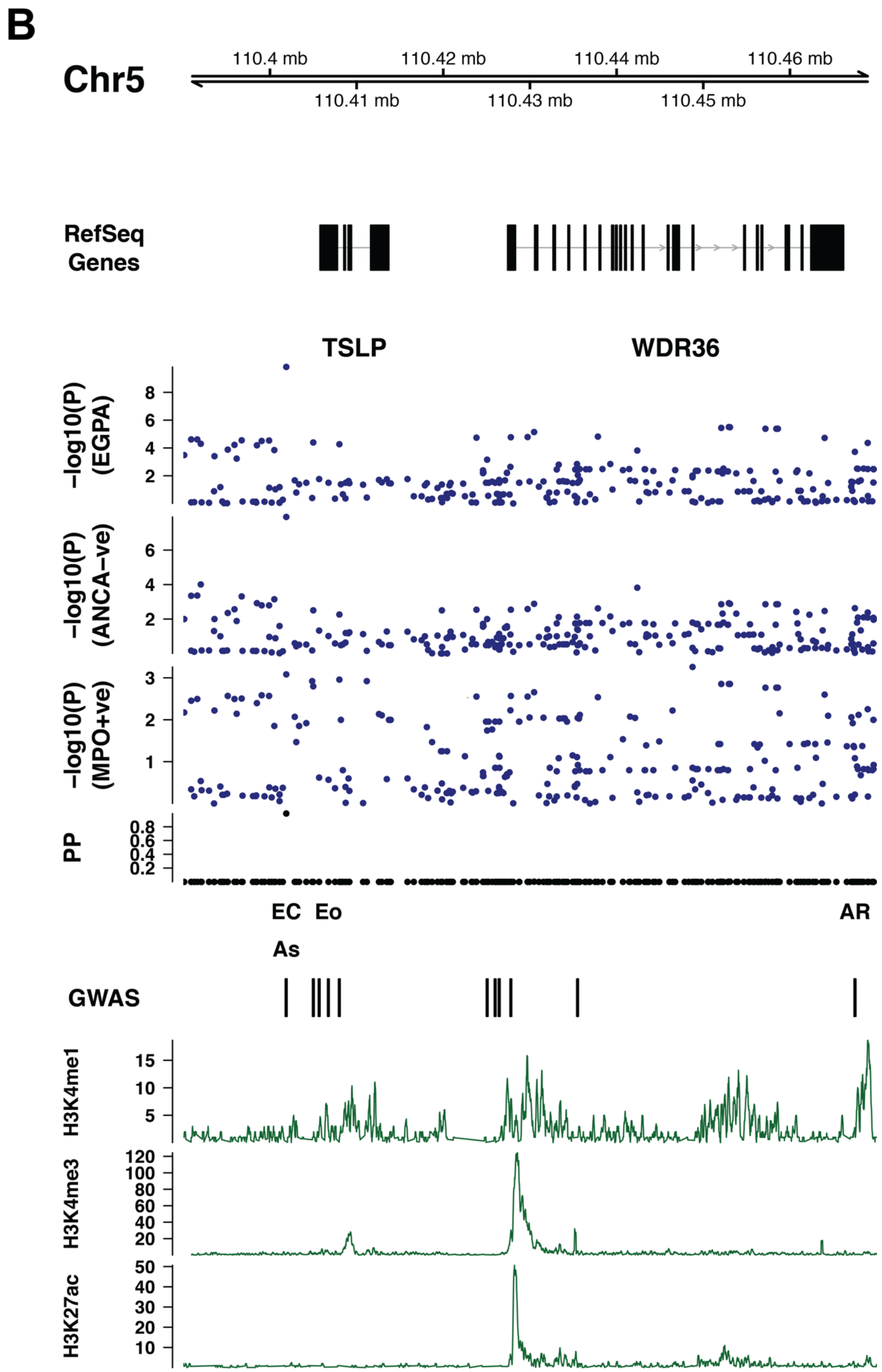
B
Chr5
110.4 mb
110.42 mb
110.44 mb
110.46 mb
110.41 mb
110.43 mb
110.45 mb
RefSeq Genes
TSLP
WDR36
−log10(P) (EGPA)
−log10(P) (ANCA−ve)
−log10(P) (MPO+ve)
PP
EC
Eo
AR
As
GWAS
H3K4me1
H3K4me3
H3K27ac

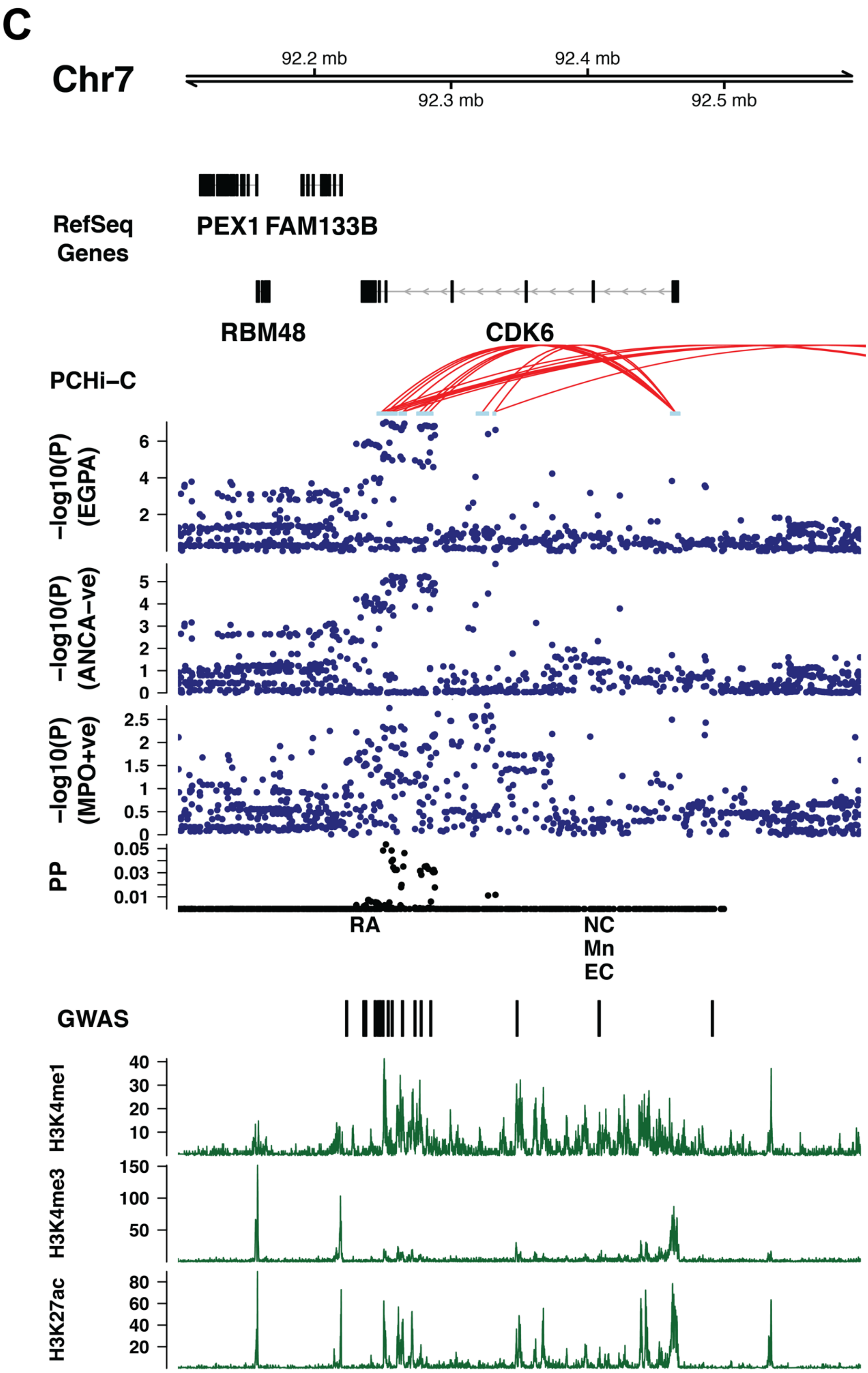
C
Chr7
92.2 mb
92.4 mb
92.3 mb
92.5 mb
RefSeq Genes
PEX1
FAM133B
RBM48
CDK6
PCHi−C
−log10(P) (EGPA)
−log10(P) (ANCA−ve)
−log10(P) (MPO+ve)
PP
RA
NC
Mn
EC
GWAS
H3K4me1
H3K4me3
H3K27ac

D

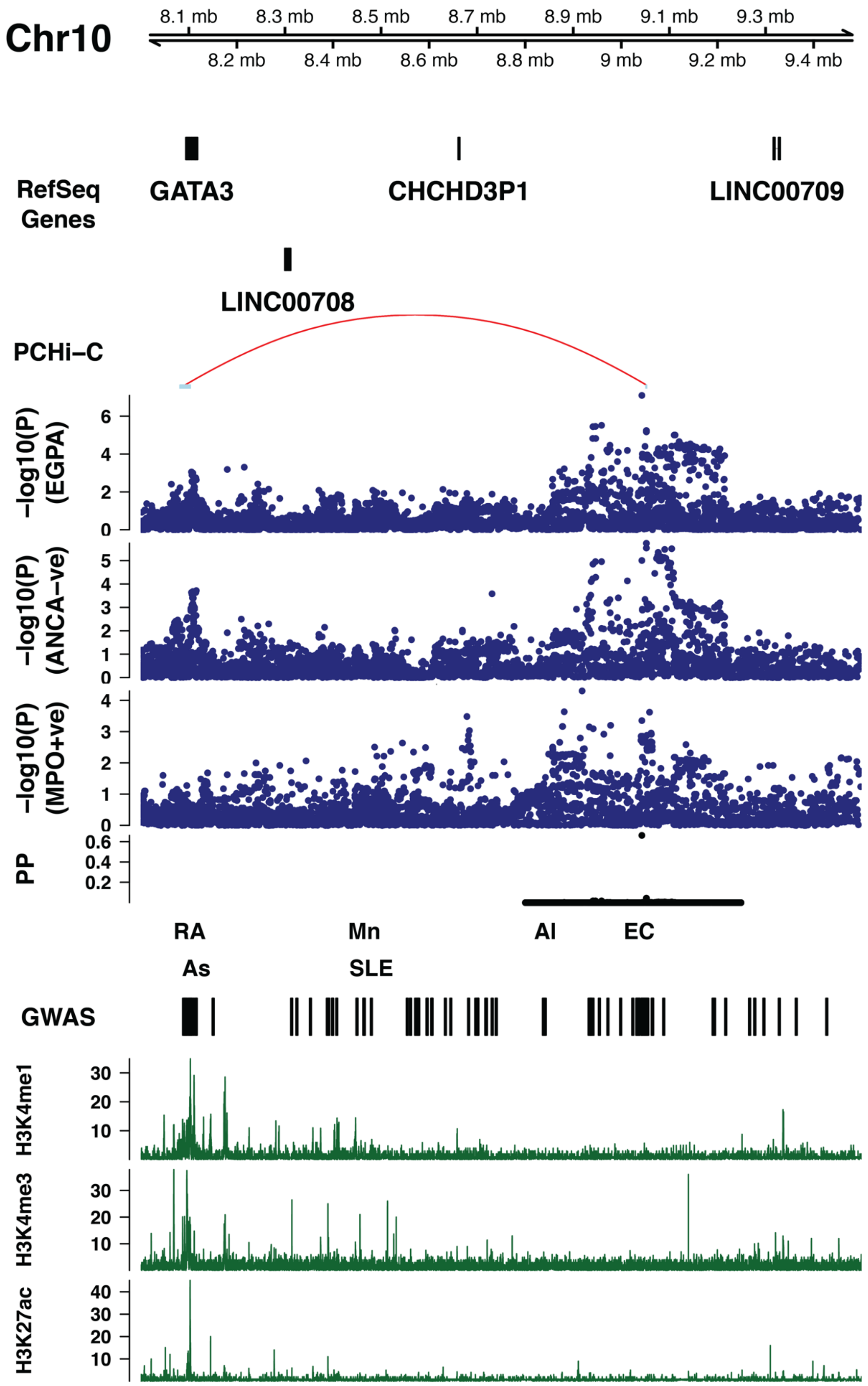
Chr10
8.1 mb
8.3 mb
8.5 mb
8.7 mb
8.9 mb
9.1 mb
9.3 mb
8.2 mb
8.4 mb
8.6 mb
8.8 mb
9 mb
9.2 mb
9.4 mb
RefSeq Genes
GATA3
CHCHD3P1
LINC00709
LINC00708
PCHi–C
–log10(P) (EGPA)
–log10(P) (ANCA–ve)
–log10(P) (MPO+ve)
PP
RA
Mn
AI
EC
As
SLE
GWAS
H3K4me1
H3K4me3
H3K27ac

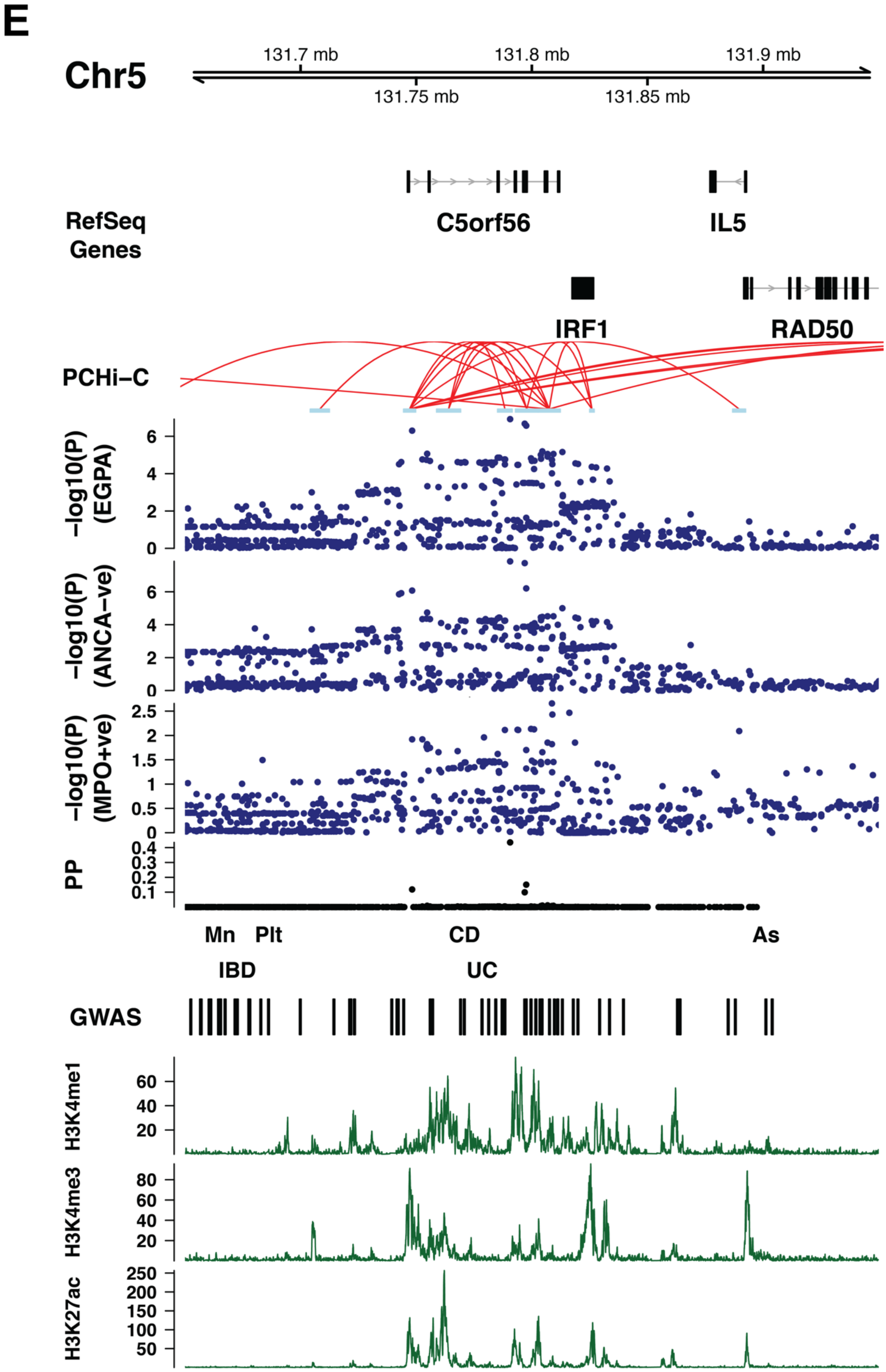
E
Chr5
131.7 mb
131.8 mb
131.9 mb
131.75 mb
131.85 mb
RefSeq Genes
C5orf56
IL5
IRF1
RAD50
PCHi−C
−log10(P) (EGPA)
−log10(P) (ANCA−ve)
−log10(P) (MPO+ve)
PP
Mn
Plt
CD
As
IBD
UC
GWAS
H3K4me1
H3K4me3
H3K27ac

F

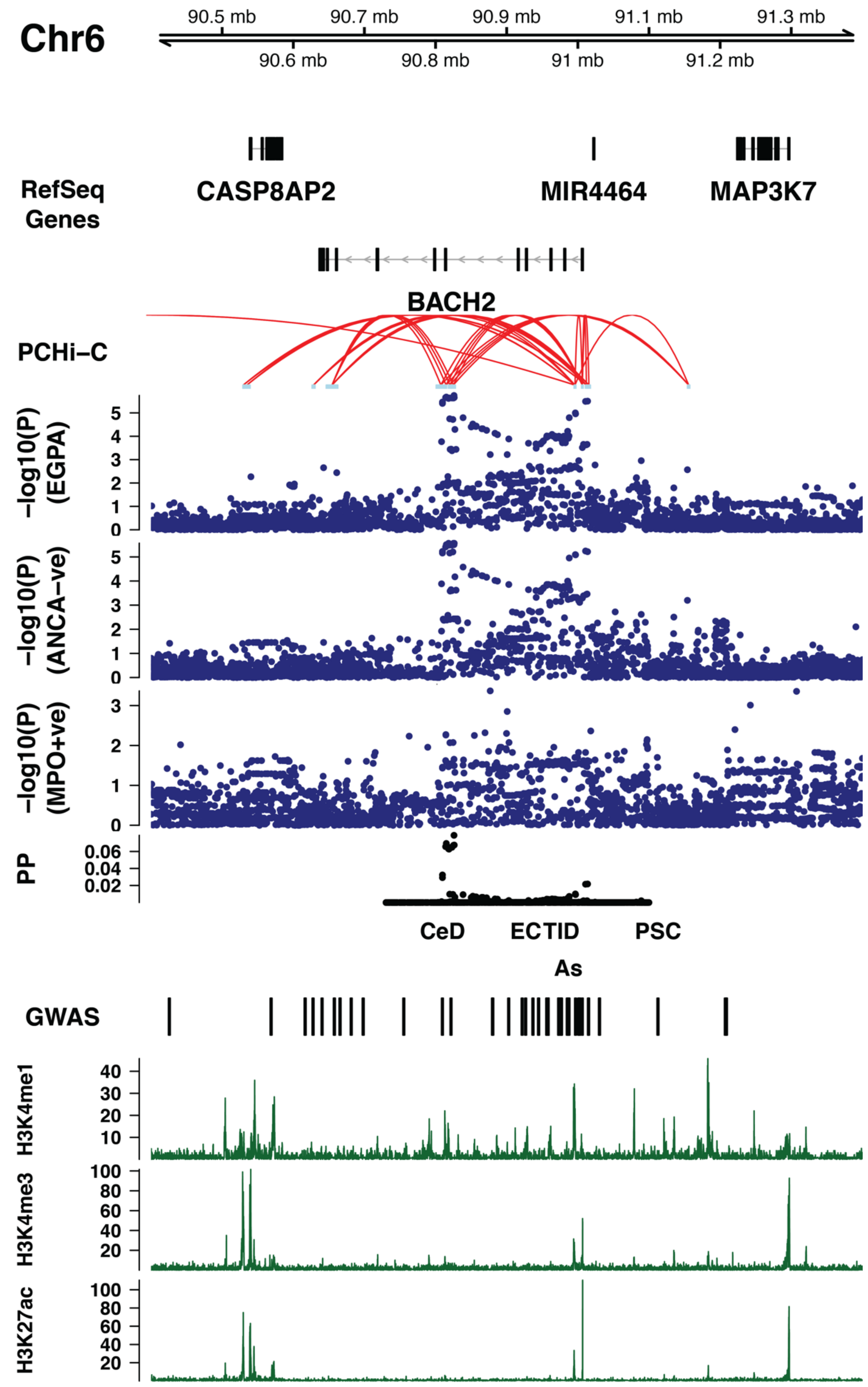
Chr6
90.5 mb
90.7 mb
90.9 mb
91.1 mb
91.3 mb
90.6 mb
90.8 mb
91 mb
91.2 mb
RefSeq Genes
CASP8AP2
MIR4464
MAP3K7
BACH2
PCHi–C
−log10(P) (EGPA)
−log10(P) (ANCA−ve)
−log10(P) (MPO+ve)
PP
CeD
ECTID
PSC
As
GWAS
H3K4me1
H3K4me3
H3K27ac

G

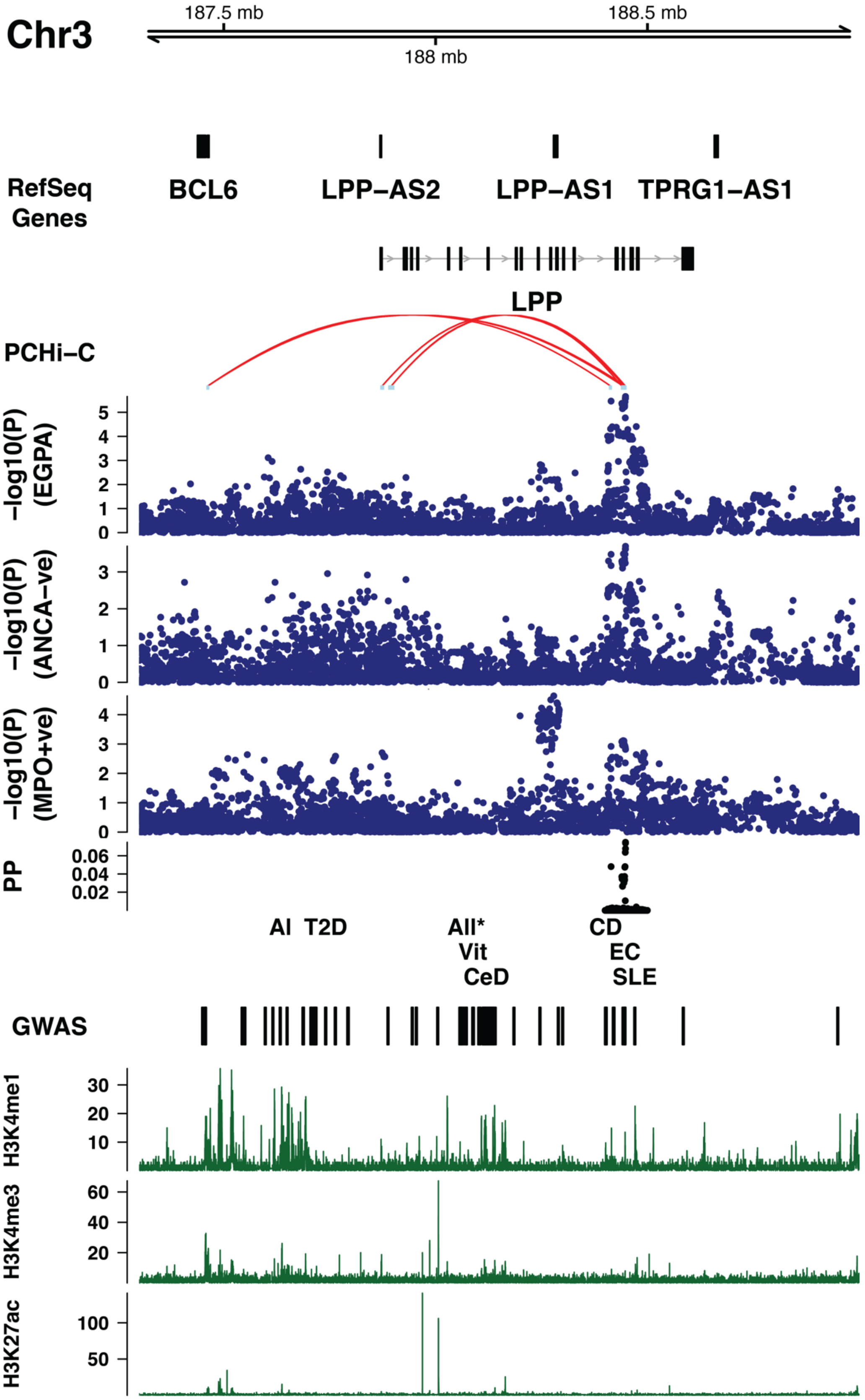
Chr3
187.5 mb
188 mb
188.5 mb
RefSeq Genes
BCL6
LPP−AS2
LPP−AS1
TPRG1−AS1
LPP
PCHi−C
−log10(P) (EGPA)
−log10(P) (ANCA−ve)
−log10(P) (MPO+ve)
PP
0.06
0.04
0.02
AI T2D
All*
Vit
CeD
CD
EC
SLE
GWAS
H3K4me1
H3K4me3
H3K27ac

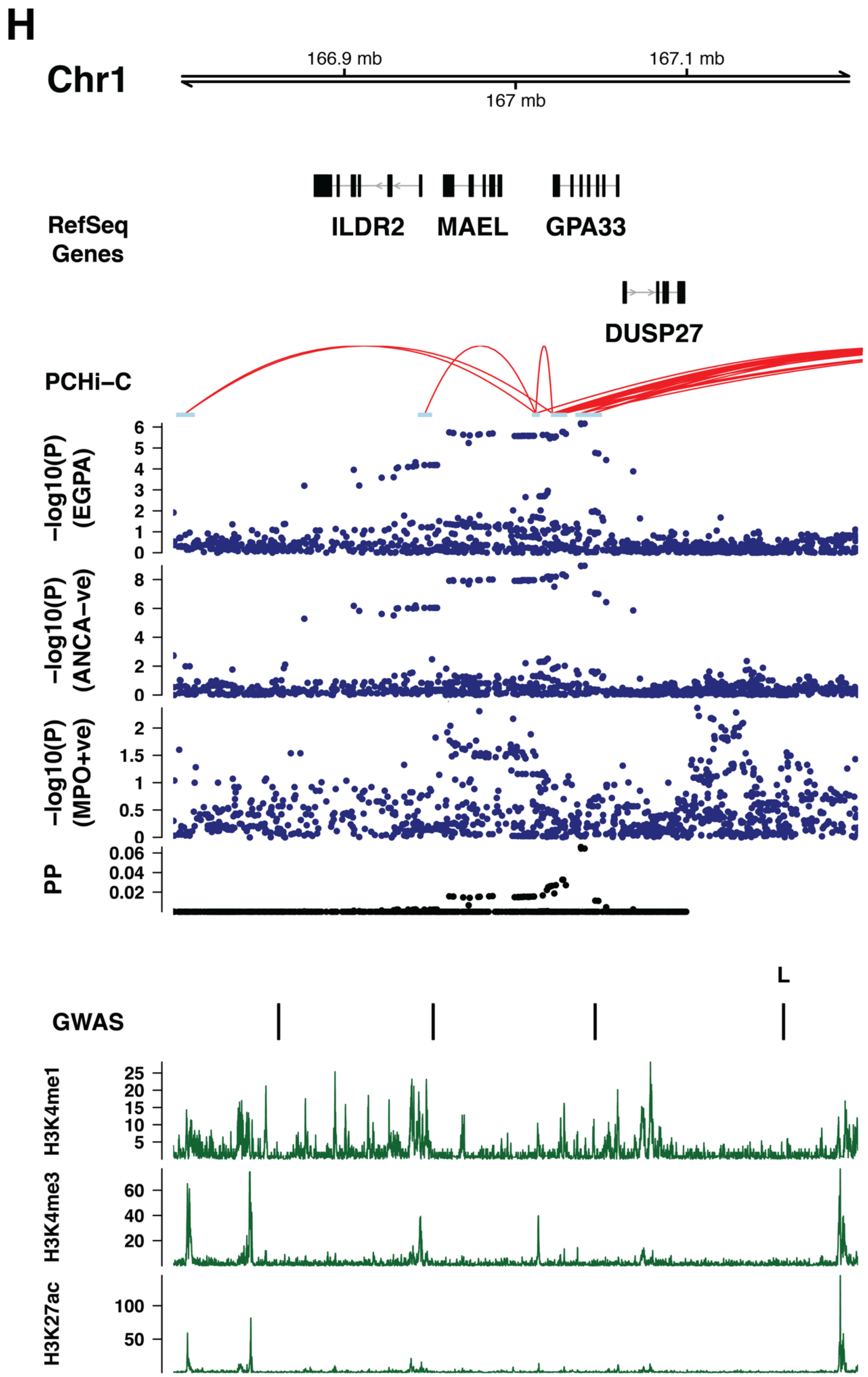
H
Chr1
166.9 mb
167 mb
167.1 mb
RefSeq Genes
ILDR2
MAEL
GPA33
DUSP27
PCHi–C
−log10(P) (EGPA)
−log10(P) (ANCA−ve)
−log10(P) (MPO+ve)
PP
GWAS
L
H3K4me1
H3K4me3
H3K27ac

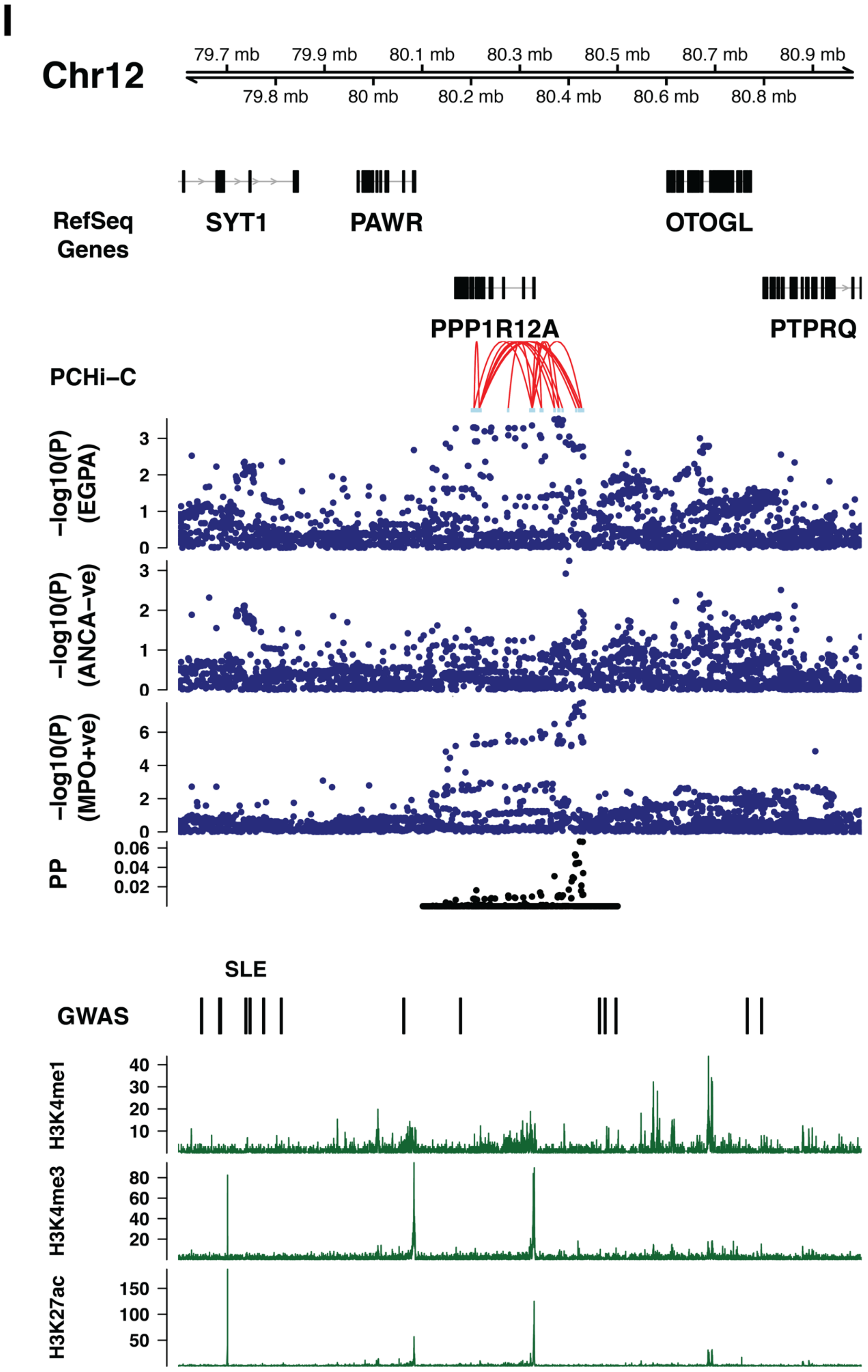
I
Chr12
79.7 mb
79.9 mb
80.1 mb
80.3 mb
80.5 mb
80.7 mb
80.9 mb
79.8 mb
80 mb
80.2 mb
80.4 mb
80.6 mb
80.8 mb
RefSeq Genes
SYT1
PAWR
OTOGL
PPP1R12A
PTPRQ
PCHi–C
−log10(P) (EGPA)
−log10(P) (ANCA−ve)
−log10(P) (MPO+ve)
PP
SLE
GWAS
H3K4me1
H3K4me3
H3K27ac

Supplementary Figure 7. Genomic features and associations with other traits at non-MHC EGPA-associated loci.

- Genomic positions from the hg19 genome build.
- The position of selected RefSeq genes
- DNA-DNA physical interactions identified by PCHi-C involving fine mapped SNPs in the indicated cell type
- Associations with EGPA as a whole, and with MPO+ and ANCA- subsets.
- PP= posterior probability from fine-mapping analysis.
- 'GWAS' panel indicates trait-associated SNPs from the NHGRI GWAS Catalog (those with p-values < 1×10^{-5}). IgAN= IgA nephropathy, RA= rheumatoid arthritis, Mn= peripheral blood monocyte count, PSC= primary sclerosing cholangitis, EC= peripheral blood eosinophil count, As= asthma, Eo= eosinophilic esophagitis, AR= allergic rhinitis, NC= neutrophil count, SLE = systemic lupus erythematosus, CD= Crohn's disease, IBD= inflammatory bowel disease, Plt= platelet count, CeD= celiac disease, T1D= type 1 diabetes, MS= multiple sclerosis, T2D= type 2 diabetes mellitus, Vit= vitiligo, All*= allergic sensitization, L = liver enzyme levels, Sz= schizophrenia.
- Histone marks from ENCODE are shown in green (GM12878 lymphoblastoid cell line). H3K4me1 = associated with enhancer function and with active transcription. H3K4me3 = marker of promoters. H3K27ac = marker of active enhancers.

(**A**) **BCL2L11 region.** PCHi-C data shown for neutrophils [10].

(**B**) **TSLP-WDR36 region.** PCHi-C data shown for neutrophils [10].

(**C**) **CDK6 region.** PCHi-C data shown for neutrophils [10].

(**D**) **10p14 intergenic region.** PCHi-C data shown for naive CD4 T cells [10].

(**E**) **C5orf56-IRF1-IL5 region.** PCHi-C data shown for neutrophils [10].

(**F**) **BACH2 region.** PCHi-C data shown for naive CD4 T cells [10].

(**G**) **LPP-BCL6 region.** PCHi-C data shown for total B cells [10].

(**H**) **GPA33 region.** PCHi-C data shown for neutrophils [10].

(I) Chr 12 intergenic region. PCHi-C shown for neutrophils [10]

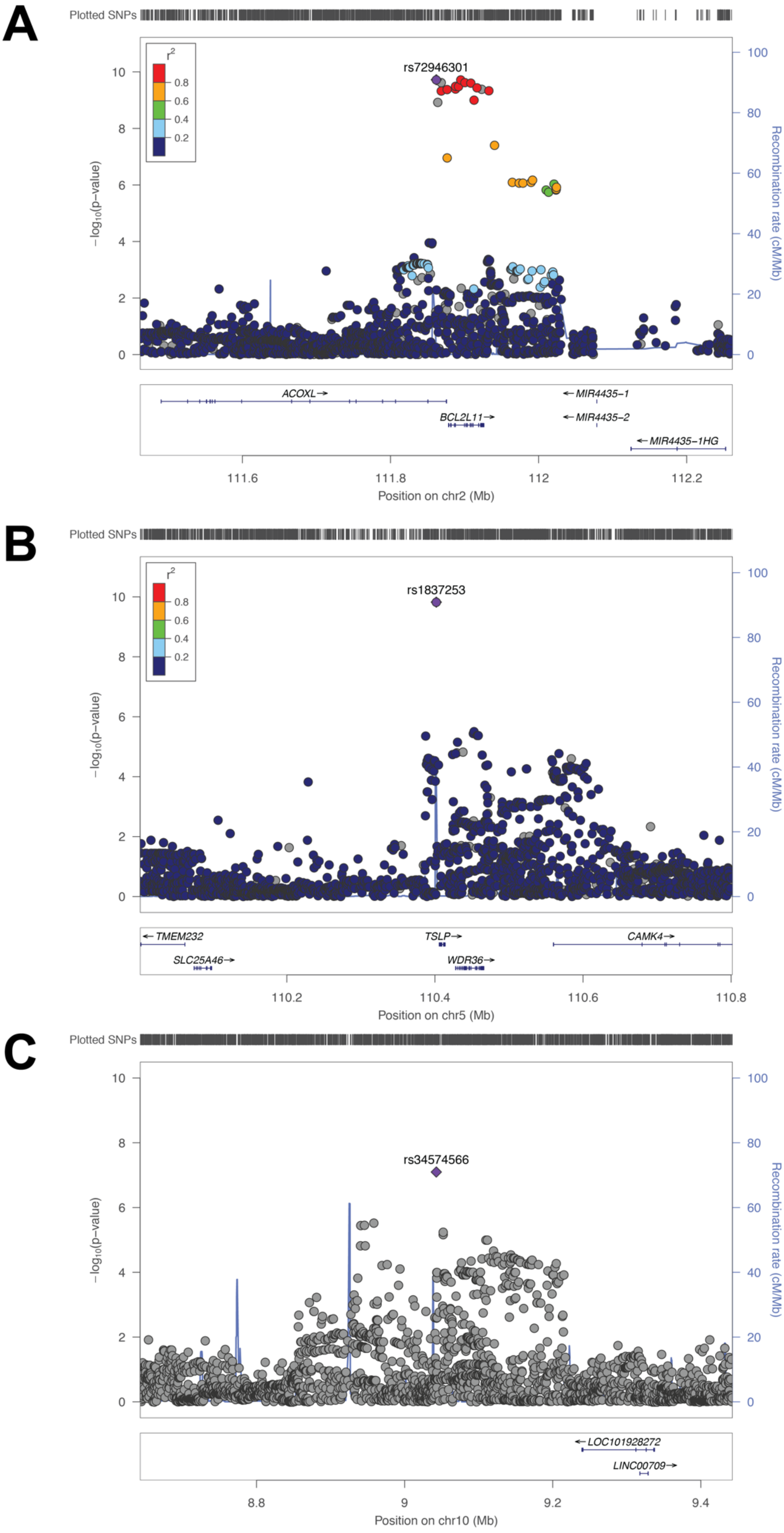
A
Plotted SNPs
r2
0.8
0.6
0.4
0.2
rs72946301
−log10(p−value)
Recombination rate (cM/Mb)
ACOXL→
BCL2L11→
←MIR4435−1
←MIR4435−2
←MIR4435−1HG
111.6
111.8
112
112.2
Position on chr2 (Mb)
B
Plotted SNPs
rs1837253
←TMEM232
SLC25A46→
TSLP→
WDR36→
CAMK4→
110.2
110.4
110.6
110.8
Position on chr5 (Mb)
C
Plotted SNPs
rs34574566
←LOC101928272
LINC00709→
8.8
9
9.2
9.4
Position on chr10 (Mb)

D

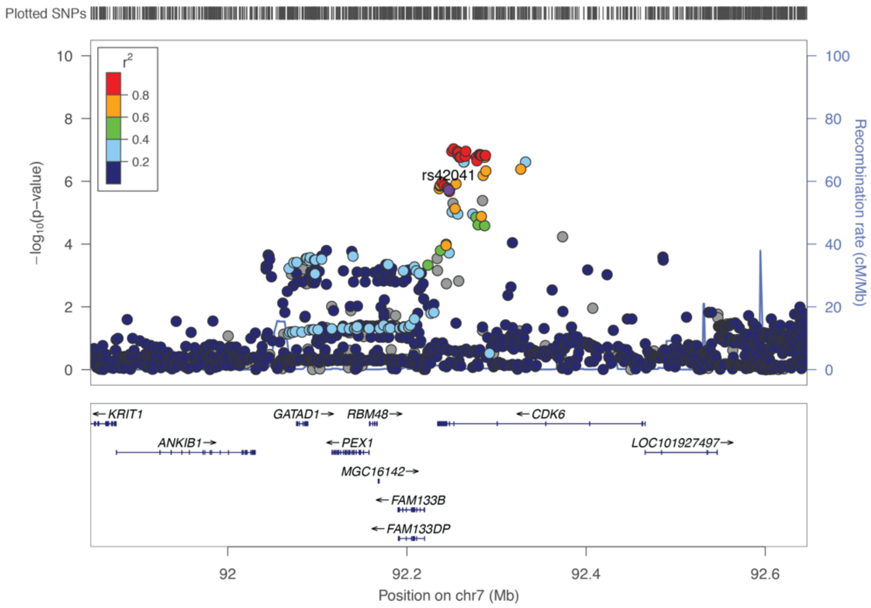
Plotted SNPs
r²
0.8
0.6
0.4
0.2
rs42041
−log10(p−value)
Recombination rate (cM/Mb)
KRIT1
ANKIB1
GATAD1
RBM48
PEX1
MGC16142
FAM133B
FAM133DP
CDK6
LOC101927497
92
92.2
92.4
92.6
Position on chr7 (Mb)

E

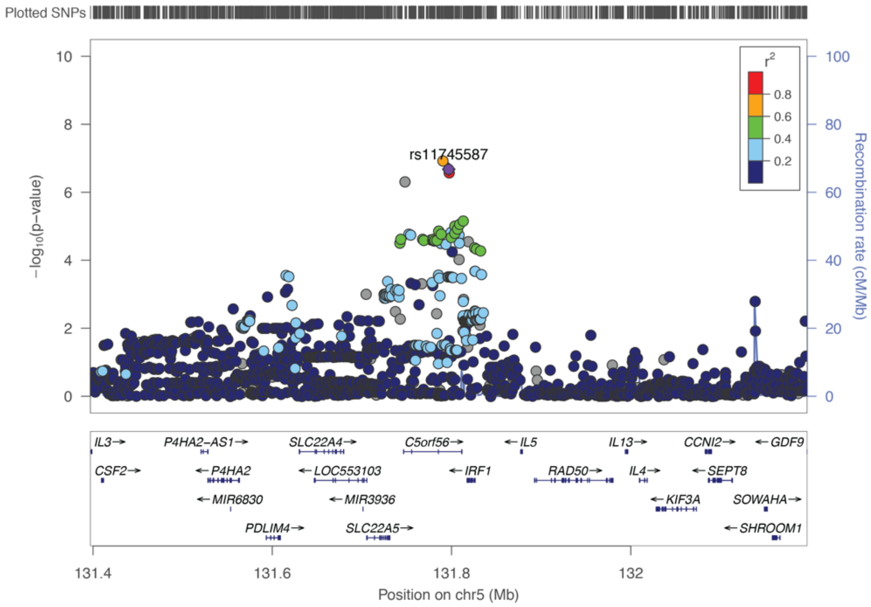
Plotted SNPs
r²
0.8
0.6
0.4
0.2
rs11745587
−log10(p−value)
Recombination rate (cM/Mb)
IL3
CSF2
P4HA2−AS1
P4HA2
MIR6830
PDLIM4
SLC22A4
LOC553103
MIR3936
SLC22A5
C5orf56
IL5
IRF1
RAD50
IL13
IL4
KIF3A
CCNI2
SEPT8
SOWAHA
GDF9
SHROOM1
131.4
131.6
131.8
132
Position on chr5 (Mb)

F

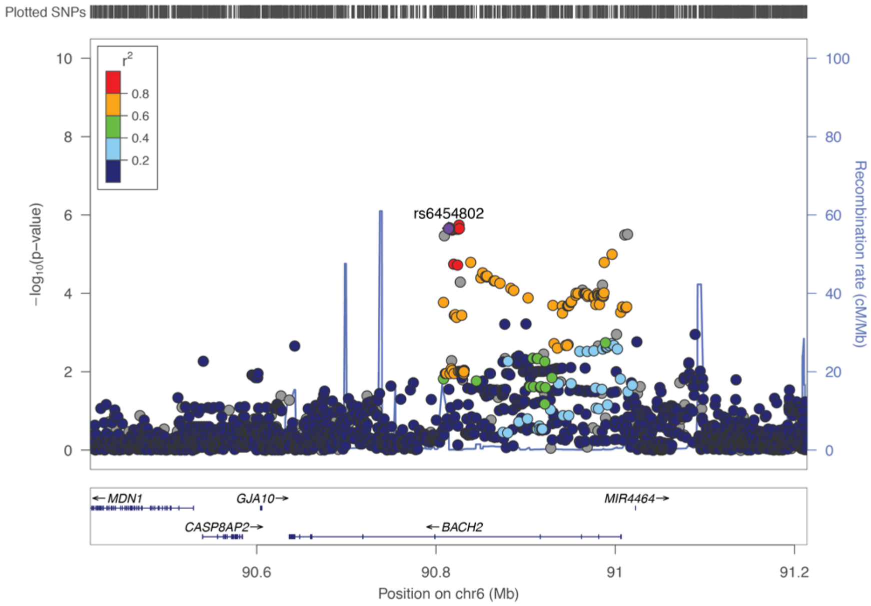
Plotted SNPs
r²
0.8
0.6
0.4
0.2
rs6454802
−log10(p−value)
Recombination rate (cM/Mb)
MDN1
GJA10
CASP8AP2
BACH2
MIR4464
90.6
90.8
91
91.2
Position on chr6 (Mb)

G

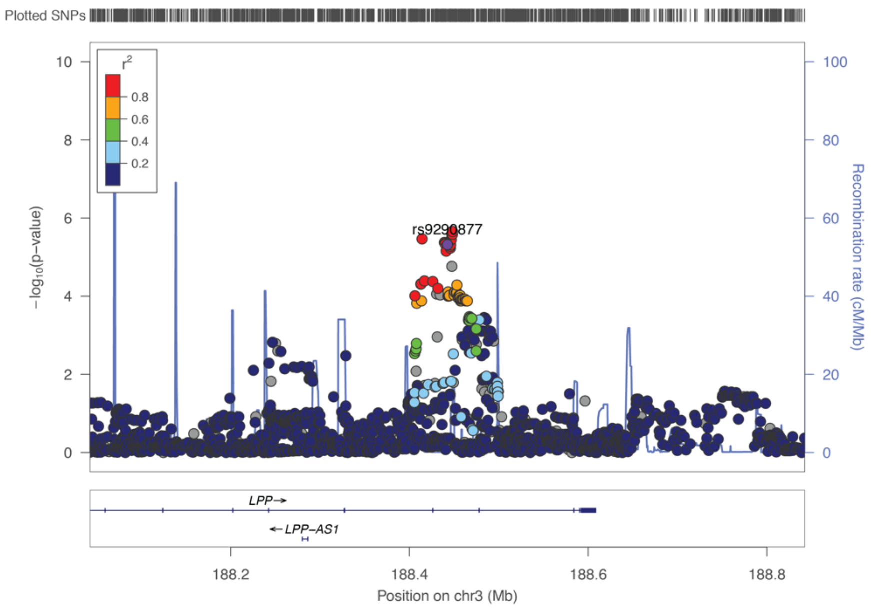
Plotted SNPs
r2
0.8
0.6
0.4
0.2
rs9290877
−log10(p−value)
Recombination rate (cM/Mb)
LPP→
←LPP−AS1
188.2
188.4
188.6
188.8
Position on chr3 (Mb)

H

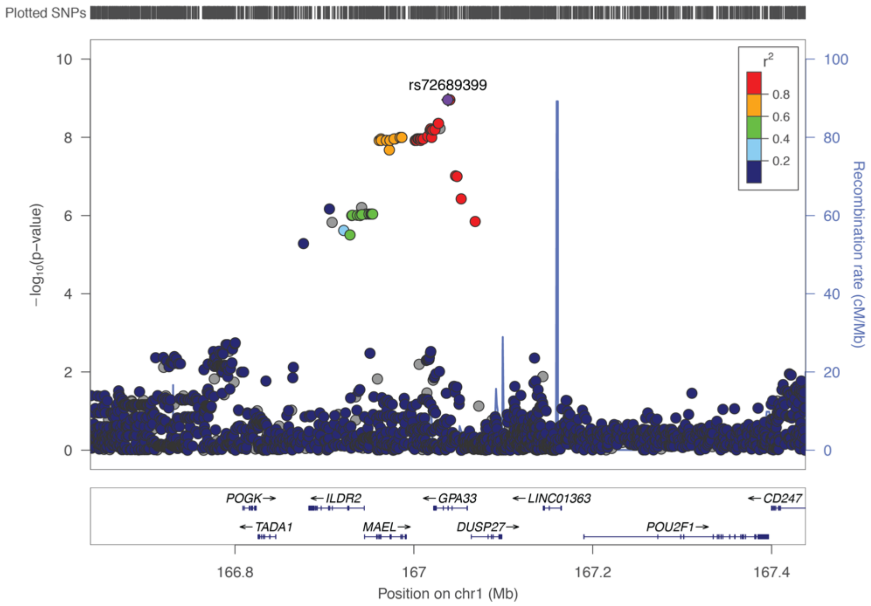
Plotted SNPs
r2
0.8
0.6
0.4
0.2
rs72689399
−log10(p−value)
Recombination rate (cM/Mb)
POGK→
←ILDR2
←GPA33
←LINC01363
←CD247
←TADA1
MAEL→
DUSP27→
POU2F1→
166.8
167
167.2
167.4
Position on chr1 (Mb)

I

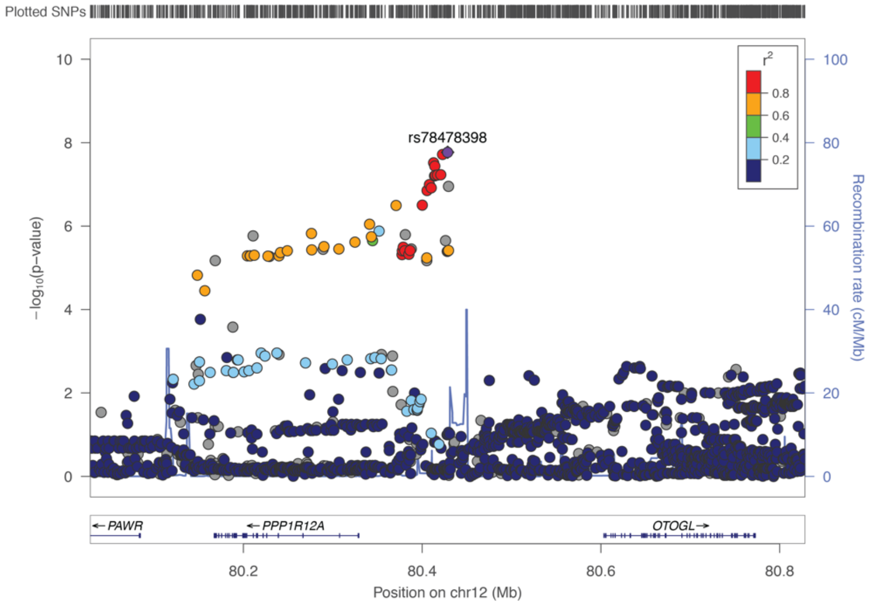
Plotted SNPs
r2
0.8
0.6
0.4
0.2
rs78478398
−log10(p−value)
Recombination rate (cM/Mb)
←PAWR
←PPP1R12A
OTOGL→
80.2
80.4
80.6
80.8
Position on chr12 (Mb)

Supplementary Figure 8. Locus zoom plots for loci associated with EGPA. Strength of association with EGPA and LD structure relative to the lead SNP at each locus. All EGPA vs controls: A) *BCL2L11*, B) *TSLP*, C) Chr 10, D) *CDK6*, E) *IRF1/IL5*, F) *BACH2*, G) *LPP*. ANCA –ve EGPA vs controls: H) *GPA33*. MPO+ vs controls: I) Chr 12 intergenic region.

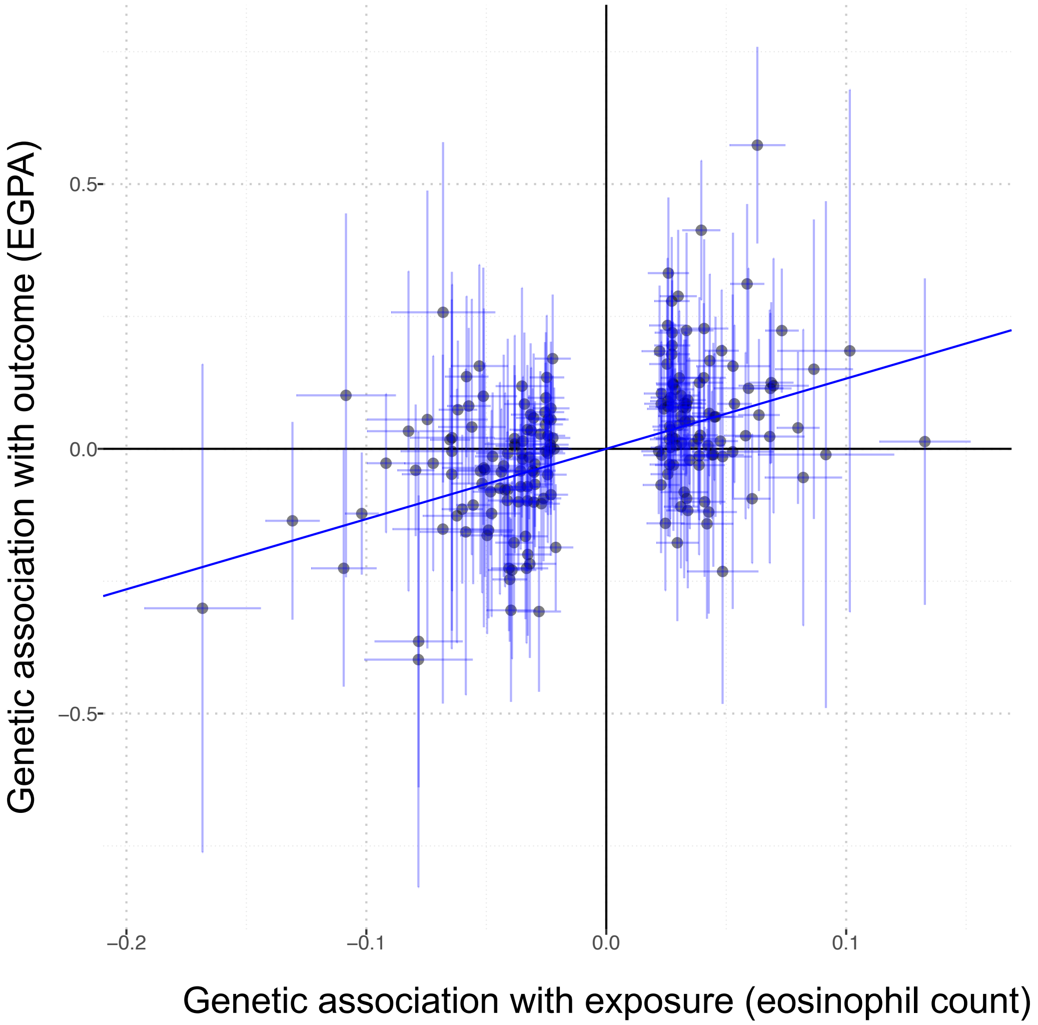

Supplementary Figure 9. Genetic effects on eosinophil count correlate with risk of EGPA. Of the 209 conditionally independent genetic variants associated with peripheral blood eosinophil count in the analysis by Astle *et al* [11], 193 (92%) were typed or imputed with INFO score >0.7 in the EGPA dataset. The point estimates for the effect sizes on eosinophil count and on EGPA risk for these 193 SNPs are shown here. Each circle represents a genetic variant. 95% confidence intervals are indicated by the horizontal and vertical bars. The effect size for eosinophil count (x-axis) is the coefficient (the 'beta') for the genotype term in the meta-analysis by Astle *et al*. The y-axis shows the estimation of the log (OR) in the EGPA GWAS. For details of conversion of BOLT-LMM beta coefficients to log(OR), see the Methods.

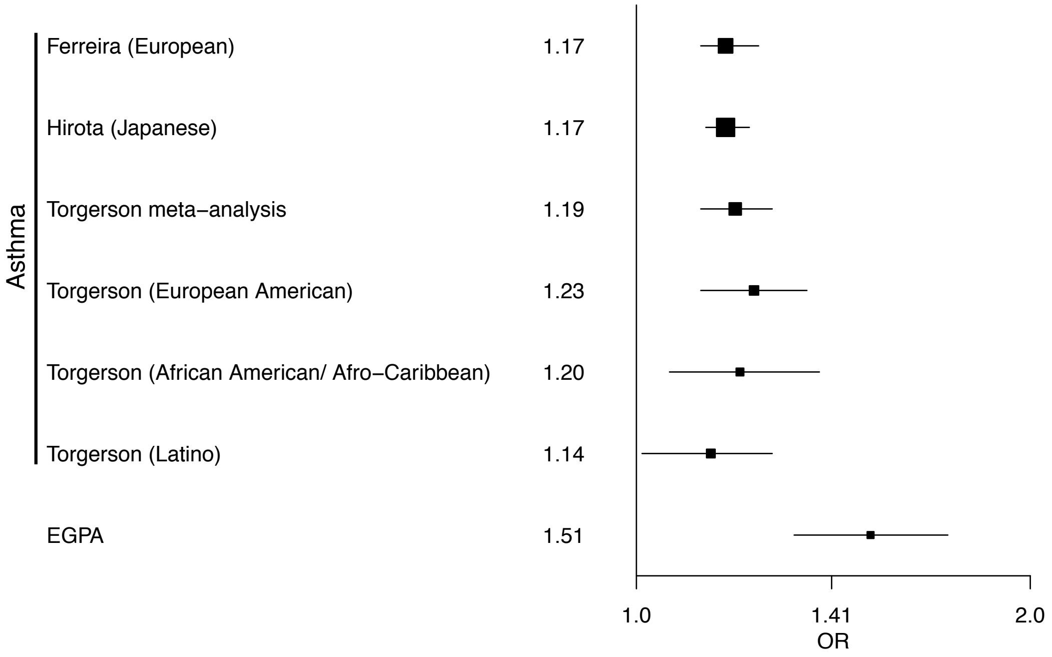

Supplementary Figure 10. The *TSLP* promoter region variant rs1837253 has a greater effect size in EGPA than in asthma. Forest plot comparing odds ratio estimates from genome-wide association studies of asthma to EGPA. Black squares indicate estimated odds ratios (also printed numerically in the column to the left of the plot). Horizontal lines indicate 95% confidence intervals. Asthma studies are indicated by the name of the first author, with the ancestry of the cohort studied in parentheses. EGPA OR and confidence intervals calculated from BOLT-LMM analysis.

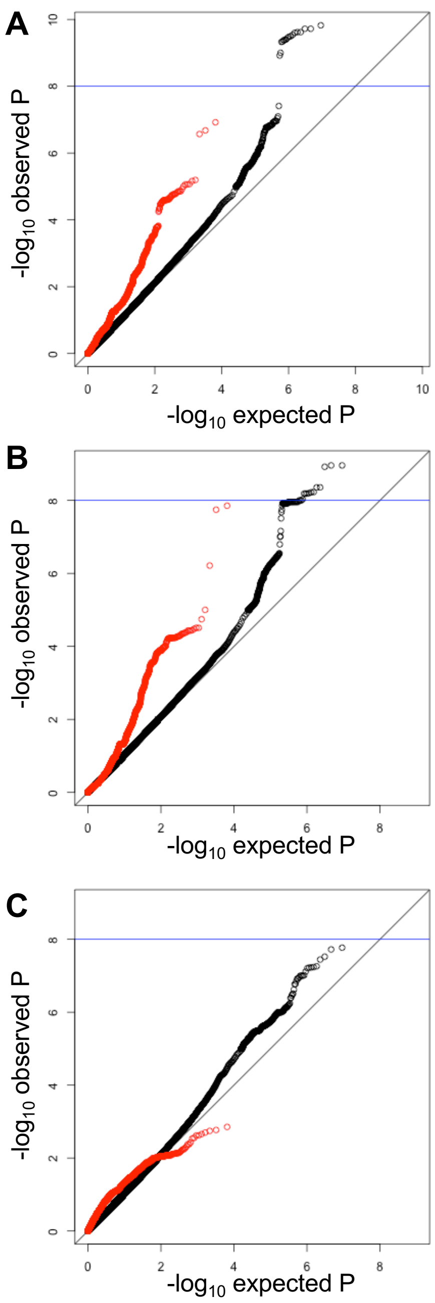

Supplementary Figure 11. QQ plots of genetic associations in EGPA according to association in IBD. A) All EGPA cases vs controls. B) ANCA –ve EGPA vs controls. C) MPO+ EGPA vs controls. Black circles indicate all genetic variants in the EGPA study. Red circles represent the subset of genetic variants with genome-wide significance (P <5x10-8) in IBD. IBD summary statistics were taken from the GWAS by Liu *et al* [12](European-ancestry individuals).

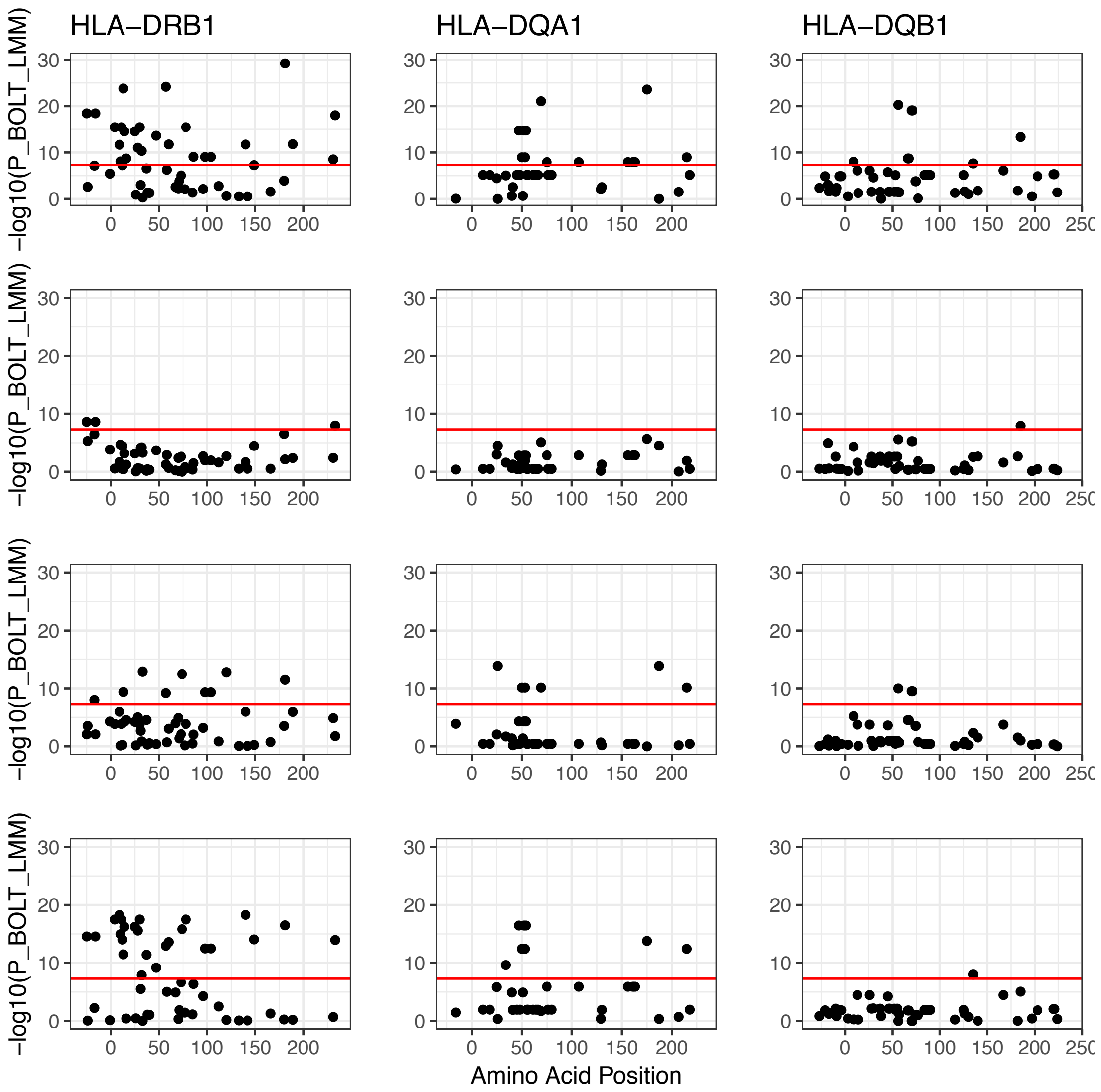

Supplementary Figure 12. Amino acid positions in HLA-DRB1, HLA-DQA1 and HLA-DQB1 associated with susceptibility to EGPA. Amino acid positions in HLA-DRB1, HLA-DQA1 and HLA-DQB1 associated with susceptibility to EGPA (A) and following conditioning on position 74 in HLA-DRB1 (B), position 175 in HLA-DQA1 (C) and position 56 in HLA-DQB1 (D).

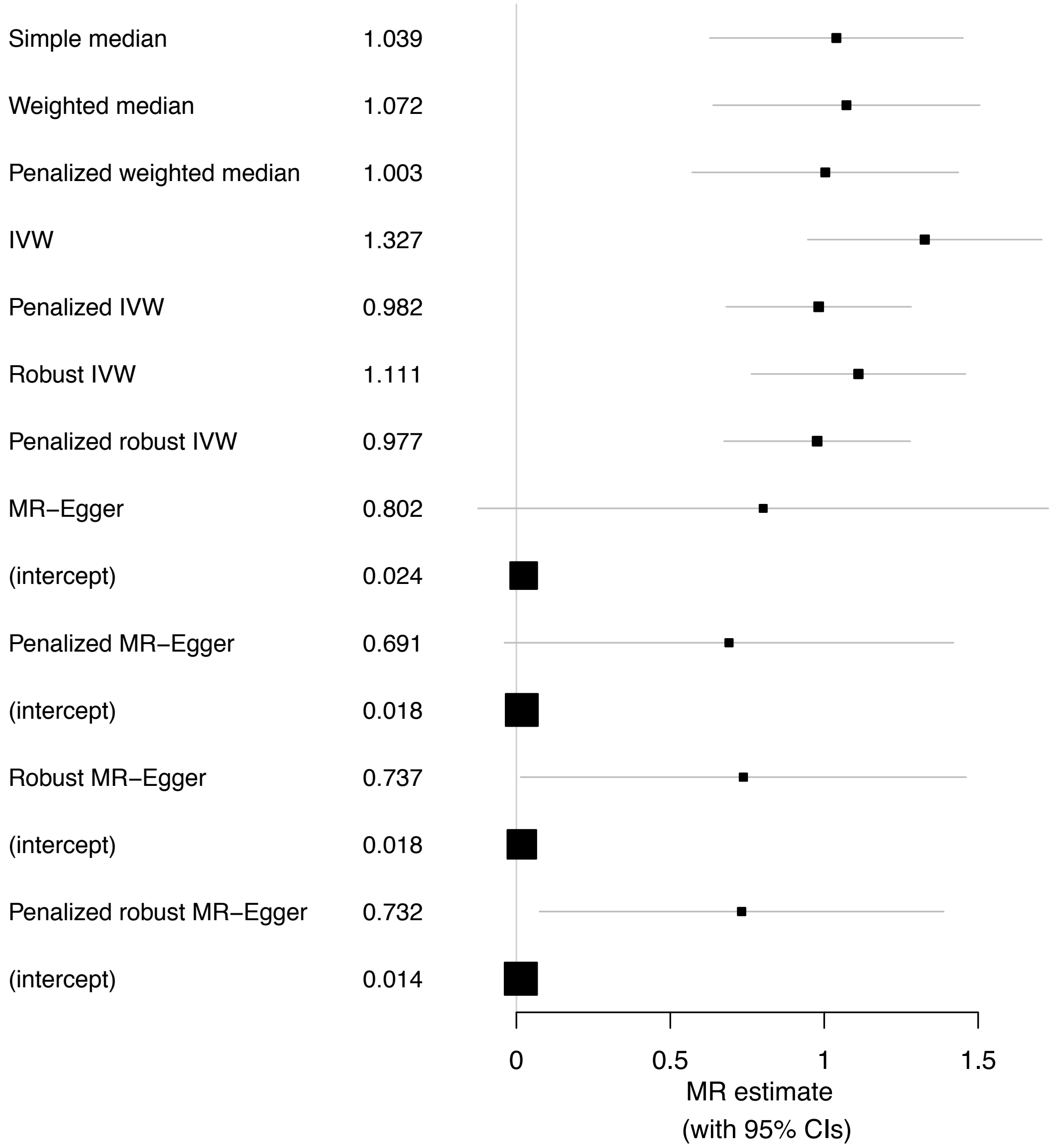

Supplementary Figure 13. Forest plot of Mendelian randomization estimates for the causal effect of eosinophil count on EGPA. Point estimates with 95% confidence intervals are shown for multiple Mendelian randomization methods. MR analysis performed using P-values for all EGPA vs controls from BOLT- LMM. IVW = inverse variance weighted.

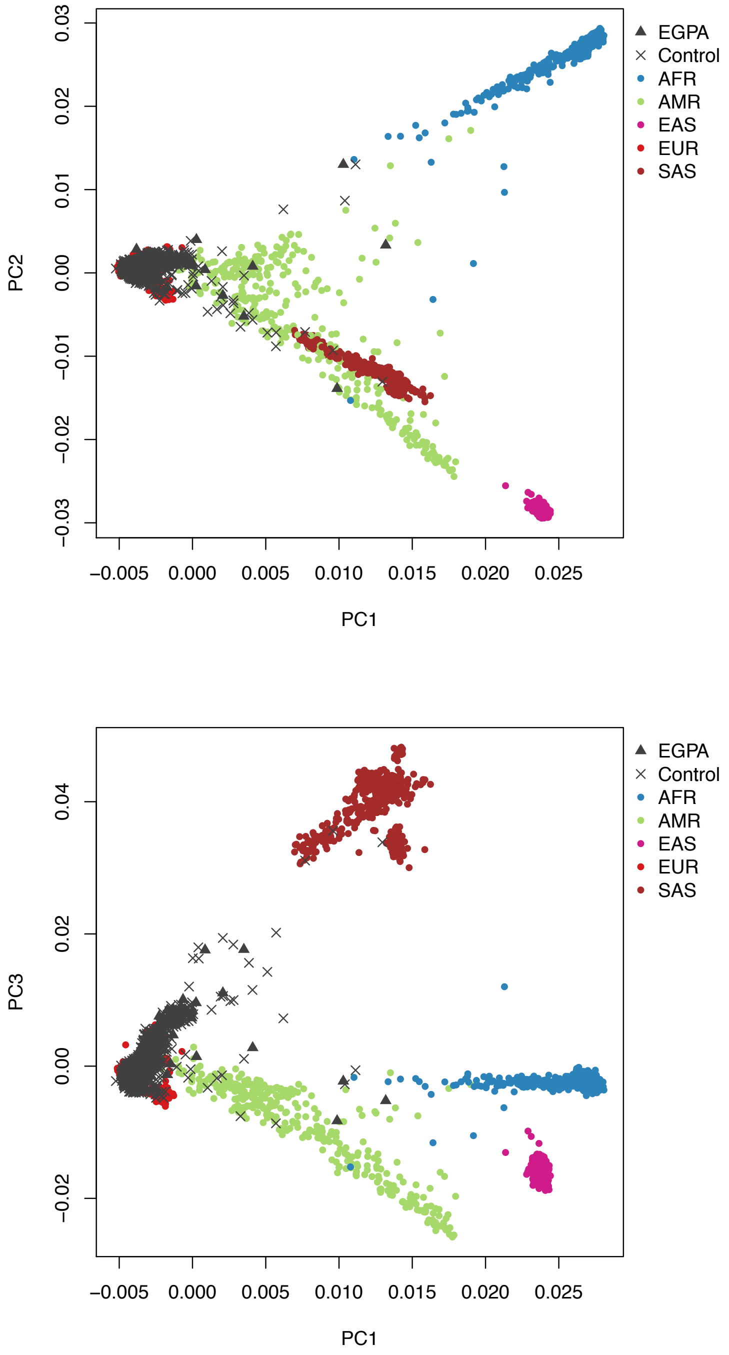

Supplementary Figure 14. Principal components analysis (PCA) of genotype data. PCA plots show ancestry of EGPA patients and controls (before removal of non-European ancestry individuals) in relation to 1000 Genomes Project individuals. EGPA patients and controls are coloured dark grey. Non-European ancestry cases and controls were removed prior to subsequent analysis.

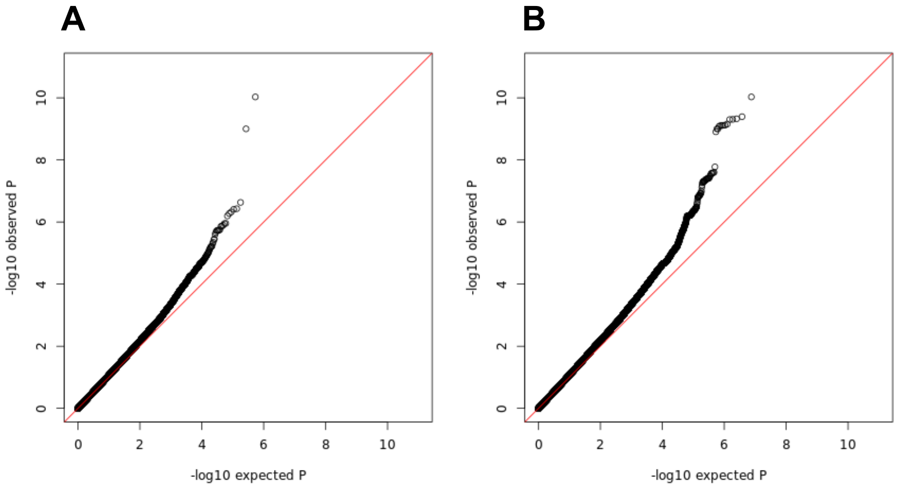

Supplementary Figure 15. QQ plots for all EGPA cases vs controls using logistic regression (SNPTEST software). (A) Directly genotyped variants. (B) Directly genotyped variants and imputed SNPs with ‘info’ metric >0.9. The line y = x is shown in red. The MHC region has been excluded.

Supplementary Tables

Supplementary Table 1. Criteria for the diagnosis of EGPA from the 'Study to Investigate Mepolizumab in the Treatment of Eosinophilic Granulomatosis With Polyangiitis' (MIRRA[$])

A diagnosis of EGPA requires both:<br>-**Asthma**<br>AND<br>-**Eosinophilia** (>1.0x10^9/L and/or >10% of total blood leucocytes)<br>PLUS at least 2 of the following additional features of EGPA:	
**Positive biopsy**	A biopsy showing histopathological evidence of eosinophilic vasculitis, or perivascular eosinophilic infiltration, or eosinophil-rich granulomatous inflammation.
**Neuropathy**	Either mononeuritis or polyneuropathy demonstrated by a motor deficit or nerve conduction abnormality
**Pulmonary infiltrates**	Non-fixed
**Sino-nasal abnormality**	
**Cardiomyopathy**	Established by echocardiography or cardiac magnetic resonance imaging
**Glomerulonephritis**	Hematuria, red cell casts, proteinuria
**Alveolar haemorrhage**	Confirmed by bronchoalveolar lavage
**Palpable purpura**	
**Positive ANCA**	Positive MPO or PR-3 ANCA

[$] https://clinicaltrials.gov/ct2/show/record/NCT02020889

Supplementary Table 2. Breakdown of 534 cases and 6688 controls by country (primary cohort).

Country	Cases	Controls
United Kingdom and Republic of Ireland	97	5465 (EPIC)
Germany	147	273
Czech Republic	5	130
Poland	48	118
France	68	0
Italy	119	266
Spain	29	93
Sweden	21	343

UK controls from the European Prospective Investigation into Cancer and Nutrition (EPIC) study.

Supplementary Table 3. Ethics approval from each contributing centre

Centre/DNA bank	Ethics number	Ethics committee
**Overarching GWAS ethics**	10/H0308/1	Cambridgeshire 2 Research Ethics Committee
**Watts DNA bank**	MREC 03/0/118	MREC for Scotland
**University of Erlangen-Nuremberg**	No. 3604	Ethics Committee of the University of Erlangen-Nuremberg
**Poland**	KBET/201/B/2011	Jagiellonian University Ethics Committee
**Klinikum Bad Bramstedt**	AZ 13-114	Ethics Committee of the University of Lübeck
**Lund University**	Dnr 2010/29	The Regional Ethical Review Board, Lund, Sweden
**University Hospital of Parma**	29932-08/10/2008	Ethics Committee of Parma University Hospital
**Paris**	2009-A01331-56	Comite de Protection des Personnes Ile de France X
**Karolinska University Hospital**	2008/1143-31	Regional Ethics Committee in Stockholm.
**Hospital Clinic Barcelona**	HCB/2016/0274	Ethics committee of the Hospital Clínic of Barcelona
**San Raffaele Hospital - Milan**	Autoimmuno-mol Protocol	Ethics Committee of San Raffaele Hospital, Milan, Italy
**Uppsala University**	2011/241/2	Regional Ethics Committee in Uppsala, Sweden
**University Hospital Prague**	1738/07 (S-IV)	Ethics Committee of the General University Hospital in Prague
**St James's Hospital, Dublin**	01/03/2010	SJH/AMNCH Research Ethics Committee

Supplementary Table 4. Direction of effect at EGPA-associated non-HLA variants on eosinophil count and asthma risk

Variant id	Position (hg19)	Typed or imputed	Variant type*	Risk allele for EGPA (major/ minor)	Effect on eosinophil count [11]	Effect on asthma risk [13]	Effect on asthma in UK Biobank
rs72946301	2:111861838	imputed	*ACOXL* intron variant; *ACOXL-AS1* (antisense RNA) non-coding transcript variant; *MIR4435-2HG* (lincRNA) non-coding variant	A (minor)	↑	na	↑†
rs9290877[$]	3:188442480	typed	*LPP* intron variant, non-coding transcript variant, upstream gene variant	C (minor)	↑	na	↑
rs1837253	5:110401872	typed	*TSLP* upstream gene variant	C (major)	↑	↑	↑
rs11745587[$]	5:131796922	typed	*C5orf56* non-coding transcript variant, intron variant, downstream gene variant; *AC116366.3* intron variant, non-coding transcript variant	A (minor)	↑	↑	↑
rs6454802[$]	6:90814199	typed	*BACH2* intron variant, non-coding transcript variant	C (major)	↑	↑	↑
rs42041[$]	7: 92246744	typed	*CDK6* intron variant, non-coding transcript variant	G (minor)	↑	na	NS
rs34574566	10:9042745	imputed	Intergenic variant	CT (major)	↑	na	na
rs72689399	1:167038121	imputed	*GPA33* intron variant, non-coding transcript variant, NMD transcript variant	T (minor)	NS	NS	NS
rs78478398	12:80428530	imputed	Intergenic variant	C (minor)	NS	NS	NS

* from Ensembl Variant Effect Predictor tool; na, variant or an LD proxy not available; NS, not significant; UTR, untranslated region; NMD, nonsense-mediated decay; [$]significant by cFDR; † P not genome-wide significant (P $7.7x10^{-5}$). Blue shading indicates the association was identified in the GWAS of ANCA –ve EGPA vs controls, pink shading in MPO +ve EGPA vs controls.

Supplementary Table 5. Replication cohort case demographics by country of origin

	Germany	Italy
Number	49	101
Gender (M/F)	16/33	43/58
ANCA -ve	42	56
ANCA +ve	6	43
MPO ANCA+ve	4	41

Supplementary Table 6. Meta-analysis of genetic associations with EGPA in the primary and replication cohorts

			Total EGPA					
			Primary cohort N = 534 cases N = 6688 controls		Replication cohort N = 142 cases N = 121 controls		Combined cohort N = 676 cases N = 6809	
Chr	SNP	Gene	P	Beta	P	Beta	P	Beta
2	rs72946301	*BCL2L11*	$1.9x10^{-10}$	0.59	0.02	0.61	$9.0x10^{-11}$	0.59
5	rs1837253	*TSLP*	$1.5x10^{-10}$	0.41	0.02	0.51	$5.2x10^{-11}$	0.42
6	rs9274704	*HLA-DQ*	$8.2x10^{-16}$	0.69	$2.5x10^{-5}$	0.80	$1.2x10^{-20}$	0.70
10	rs34574566	*10p14*	$8.0x10^{-8}$	-0.36	0.11	-0.33	$2.9x10^{-8}$	-0.36

Supplementary Table 7: ANCA status according to country of recruitment

Countries	ANCA negative (N)	MPO positive (N)	Total*	% MPO positive
Czech Republic	2	3	5	60.0
France	48	19	68	27.9
Germany	124	22	147	15.0
Italy	56	57	119	47.9
Poland	36	5	48	10.4
Spain	12	16	29	55.2
Sweden	12	9	21	42.9
UK & Republic of Ireland	62	28	97	28.9

*total is greater than sum of MPO+ and ANCA –ve because of patients with PR3 ANCA or positive ANCA immunofluorescence without MPO or PR3 antibodies

Supplementary Table 8: associations of ANCA status with clinical features using logistic regression with adjustment for country of origin.

Clinical feature	*Nominal P value*	*Bonferroni adjusted P value*	*Odds ratio (95% CI)*
**Neuropathy**	**$2.29x10^{-5}$**	**$1.83x10^{-4}$**	**2.69 (1.70, 4.26)**
**Lung infiltrates**	**0.0029**	**0.023**	**0.51 (0.33, 0.80)**
ENT	0.41	1.0	0.80 (0.46, 1.37)
**Cardiomyopathy**	**0.00055**	**$4.36x10^{-3}$**	**0.39 (0.23, 0.67)**
**Glomerulonephritis**	**$3.85x10^{-6}$**	**$3.08x10^{-5}$**	**3.87 (2.18, 6.87)**
Lung haemorrhage	0.47	1.0	1.46 (0.52, 4.08)
Purpura	0.15	1.0	0.70 (0.43, 1.13)
Positive biopsy	0.52	1.0	0.87 (0.57, 1.33)

Analysis confined to ANCA –ve and MPO ANCA +ve patients (n = 352+159= 511). Patients who were PR3 ANCA positive, or who were ANCA positive by immunofluorescence without MPO or PR3 antibodies were excluded. Odds ratios are for MPO autoantibody positivity (i.e. positive odds ratios indicate increased prevalence of the clinical feature in MPO antibody positive cases). Significant associations are highlighted in bold.

Supplementary Table 9. Meta-analysis of genetic associations with EGPA subsets in the primary and replication cohorts

EGPA subset				Primary cohort		Replication cohort		Combined cohort	
	Chr	Gene	SNP	P	Beta	P	Beta	P	Beta
MPO +ve EGPA*									
	6	*HLA-DQ*	rs17212014	$5.9x10^{-34}$	2.15	$1.2x10^{-7}$	1.53	$1.1x10^{-41}$	2.0
	12	*12q21*	rs78478398	$1.7x10^{-8}$	1.87	0.25	0.71	$1.3x10^{-8}$	1.62
ANCA –ve EGPA^									
	1	*GPA33*	rs72689399	$1.1x10^{-9}$	1.86	0.27	1.06	$7.4x10^{-10}$	1.78
	6	*HLA*	rs6931740	$4.2x10^{-8}$	0.56	0.03	0.52	$3.4x10^{-9}$	0.55

* MPO +ve EGPA, primary cohort N = 159, replication cohort N = 43, combined cohort N = 202
^ ANCA –ve EGPA, primary cohort N = 352, replication cohort N = 94, combined cohort N = 446

Supplementary Table 10. Evidence to support biological plausibility of EGPA-associated variants

Chr	Variant	Other relevant traits with GWAS significant signals in the region*	Candidate gene(s)	eQTL or pQTL for candidate gene*?	Strength of additional evidence	Experimental data	Other
1	rs72689399	-	*GPA33*	Yes	circumstantial	GPA33 plays a role in maintaining epithelial barrier function; KO mice exhibit increased intestinal permeability and increased severity of DSS-induced colitis [14]	
2	rs72946301	EC, PSC<br><br>'Suggestive' GWAS signal for asthma in UK Biobank (P 9.9×10^{-6} for rs72836344 (LD proxy variant, r2 0.93)	*BCL2L11*	Yes	strong	KO mouse: defective apoptosis of immune cells; autoimmunity [15-18]	
			MORRBID	No	strong	MORRBID KO: deficient in eosinophils. [19]<br><br>MORRBID regulates myeloid cell survival via BCL2L11 expression [19]<br><br>MORRBID expression higher in HES patients cf controls. [19]<br><br>MORRBID expression correlates with IL5 expression. [19]	
			ACOXL	No	-	-	
3			*LPP*	Yes	-	-	

	rs9290877	EC, Asthma, Allergic disease	*BCL6*	No	strong	BCL6-deficient mice die of overwhelming eosinophilic inflammation characterized by myocarditis and pulmonary vasculitis [20]	GWAS signals for allergy and immune-mediated diseases in this region at variants independent of EGPA hit
5	rs1837253	EC,<br>Asthma,<br>Nasal polyps,<br>Combined asthma & hayfever,<br>Allergic disease	*TSLP*	Yes[21]	strong	TSLP drives eosinophilia and enhanced TH2 responses through effects on mast cells, group 2 innate lymphoid cells (ILC2), and dendritic cells	Drugs targeting TSLP in development for asthma [22,23]<br><br>GWAS signals for other eosinophilic diseases in this region at variants independent of EGPA hit [24-31]
5	rs11745587	EC,<br>Asthma,<br>Hayfever,<br>allergic rhinitis or eczema,<br>IBD,<br>JIA<br><br>QTL for plasma tryptophan levels$	*IRF1*	Yes	intermediate	Important immune transcriptional regulator	
			IL5	No	strong	Archetypal "eosinophilic" cytokine [32,33]	RCT evidence for anti-IL5 therapy in EGPA and eosinophilic asthma [8,34]
			C5orf56	Yes	no	-	
			IL4	No	strong		
6	rs6454802	EC<br>Asthma<br>Nasal polyps<br>PSC<br>Hayfever, allergic rhinitis or eczema	*BACH2*	Yes	strong	In B cells, BACH2 represses the transcriptional regulator BLIMP1. [35]<br><br>BACH2 also influences multiple facets of T cell differentiation and activity [36,37]	

						BACH2 deficient mice die of eosinophilic pneumonitis [38]	
7	rs42041	EC<br>Neutrophil count	*CDK6*	Yes	intermediate	Plays a role in cell cycle regulation	
10	rs34574566	EC<br>Asthma<br>Hayfever, Allergic rhinitis or eczema	*GATA3*	No	strong	GATA3 activation is a key event for Th2 cell differentiation and development [39-42]).<br><br>Directly binds Th2 locus genes and drives pro-eosinophilic cytokines [42]<br><br>GATA3 overexpression leads to eosinophilia in mice [43]<br><br>GATA3 important for development and function of ILC2 cells [44]and invariant NKT cells [45]	
12	rs78478398	-	*PPP1R12A*	No	-	-	-
			OTOGL	Yes	-	-	-

Abbreviations: EC eosinophil count, IBD inflammatory bowel disease, JIA juvenile idiopathic arthritis, PSC primary sclerosing cholangitis
* for comprehensive listings of disease associations and eQTLs that overlap with the EGPA-associated variants or their LD proxies, see Supplemental Data Items 1 and 2
$Tryptophan supplements have been linked to eosinophilic syndromes.

Supplementary Table 11: Association of classical MHC alleles with MPO+ve EGPA

MHC Allele	Unconditioned		Conditioned on					
			*DRB1*08:01*		*DRB1*08:01*<br>*DQA1*02:01*		*DRB1*08:01*<br>*DQA1*02:01*<br>*DRB1*01:03*	
	OR	P	OR	P	OR	P	OR	P
*HLA-DRB1*08:01*	35.8	$7.6x10^{-24}$	-	-	-	-	-	-
*HLA-DQA1*04:01*	26.6	$1.7x10^{-21}$	1.1	0.90	1.4	0.84	1.23	0.93
*HLA-DQB1*04:02*	24.6	$5.2x10^{-21}$	1.3	0.96	1.8	0.57	1.64	0.69
*HLA-DQA1*02:01*	4.8	$1.8x10^{-15}$	5.1	$3.5x10^{-16}$	-	-	-	-
*HLA-DRB1*07:01*	4.7	$2.7x10^{-15}$	5.1	$4.8x10^{-16}$	1.1	0.98	1.09	0.99
*HLA-DQB1*03:03*	4.5	$1.9x10^{-7}$	5.3	$7.9x10^{-9}$	2.6	$3.3x10^{-3}$	2.71	0.002
*HLA-DQA1*05:01*	0.4	$1.2x10^{-8}$	0.5	$3.2x10^{-6}$	0.6	$2.0x10^{-3}$	0.63	$9.0x10^{-4}$
*HLA-DQB1*02:02*	3.4	$2.3x10^{-8}$	3.4	$3.8x10^{-8}$	0.5	0.13	0.48	0.097
*HLA-DRB1*01:03*	11.4	$7.5x10^{-7}$	13.7	$5.7x10^{-8}$	14.0	$4.2x10^{-8}$	-	-

Supplementary Table 12. Minor allele frequencies at HLA alleles associated with EGPA stratified by country

HLA allele		Total cohort		Czech		UK		Germany		Italy		Poland		Spain		Sweden	
		Case	Cont	Case	Cont	Case	Cont	Case	Cont	Case	Cont	Case	Cont	Case	Cont	Case	Cont
HLA DQA1	02:01	0.19	0.13	0.30	0.15	0.23	0.14	0.13	0.09	0.22	0.13	0.16	0.17	0.29	0.16	0.14	0.07
	04:01	0.07	0.02	0.10	0.02	0.04	0.02	0.06	0.02	0.09	0.02	0.05	0.03	0.10	0.03	0.12	0.04
HLA DQB1	02:02	0.13	0.10	0.10	0.08	0.17	0.10	0.09	0.07	0.17	0.10	0.13	0.12	0.22	0.14	nd	0.05
	04:02	0.07	0.02	0.10	0.02	0.04	0.02	0.06	0.02	0.09	0.03	0.05	0.03	0.10	0.03	0.12	0.04
	03:03	0.06	0.05	0.20	0.07	0.08	0.05	0.04	0.04	0.05	0.04	0.04	0.05	0.07	0.02	0.19	0.05
HLA DRB1	01:03	0.02	0.01	nd	0.01	0.03	0.01	0.01	0.004	0.02	0.004	nd	nd	0.07	0.005	0.02	0.004

nd, Not detected

Supplementary references

1. Jennette, J.C. *et al.* 2012 revised International Chapel Hill Consensus Conference Nomenclature of Vasculitides. *Arthritis Rheum* **65**, 1-11 (2013).
2. Churg, J. & Strauss, L. Allergic granulomatosis, allergic angiitis, and periarteritis nodosa. *Am J Pathol* **27**, 277-301 (1951).
3. Reid, A.J. *et al.* Churg-Strauss syndrome in a district hospital. *QJM* **91**, 219-29 (1998).
4. Lanham, J.G., Elkon, K.B., Pusey, C.D. & Hughes, G.R. Systemic vasculitis with asthma and eosinophilia: a clinical approach to the Churg-Strauss syndrome. *Medicine (Baltimore)* **63**, 65-81 (1984).
5. Masi, A.T. *et al.* The American College of Rheumatology 1990 criteria for the classification of Churg-Strauss syndrome (allergic granulomatosis and angiitis). *Arthritis Rheum* **33**, 1094-100 (1990).
6. Khoury, P. *et al.* Serum biomarkers are similar in Churg-Strauss syndrome and hypereosinophilic syndrome. *Allergy* **67**, 1149-56 (2012).
7. Rao, J.K., Allen, N.B. & Pincus, T. Limitations of the 1990 American College of Rheumatology classification criteria in the diagnosis of vasculitis. *Ann Intern Med* **129**, 345-52 (1998).
8. Wechsler, M.E. *et al.* Mepolizumab or Placebo for Eosinophilic Granulomatosis with Polyangiitis. *N Engl J Med* **376**, 1921-1932 (2017).
9. Lyons, P.A. *et al.* Genetically distinct subsets within ANCA-associated vasculitis. *N Engl J Med* **367**, 214-23 (2012).
10. Javierre, B.M. *et al.* Lineage-Specific Genome Architecture Links Enhancers and Non-coding Disease Variants to Target Gene Promoters. *Cell* **167**, 1369-1384 e19 (2016).
11. Astle, W.J. *et al.* The Allelic Landscape of Human Blood Cell Trait Variation and Links to Common Complex Disease. *Cell* **167**, 1415-1429 e19 (2016).
12. Liu, J.Z. *et al.* Association analyses identify 38 susceptibility loci for inflammatory bowel disease and highlight shared genetic risk across populations. *Nat Genet* **47**, 979-86 (2015).
13. Moffatt, M.F. *et al.* A large-scale, consortium-based genomewide association study of asthma. *N Engl J Med* **363**, 1211-21 (2010).
14. Williams, B.B. *et al.* Glycoprotein A33 deficiency: a new mouse model of impaired intestinal epithelial barrier function and inflammatory disease. *Dis Model Mech* **8**, 805-15 (2015).
15. Alfredsson, J., Puthalakath, H., Martin, H., Strasser, A. & Nilsson, G. Proapoptotic Bcl-2 family member Bim is involved in the control of mast cell survival and is induced together with Bcl-XL upon IgE-receptor activation. *Cell Death Differ* **12**, 136-44 (2005).
16. Bouillet, P. *et al.* Proapoptotic Bcl-2 relative Bim required for certain apoptotic responses, leukocyte homeostasis, and to preclude autoimmunity. *Science* **286**, 1735-8 (1999).
17. Bouillet, P. *et al.* BH3-only Bcl-2 family member Bim is required for apoptosis of autoreactive thymocytes. *Nature* **415**, 922-6 (2002).

18. Enders, A. *et al.* Loss of the pro-apoptotic BH3-only Bcl-2 family member Bim inhibits BCR stimulation-induced apoptosis and deletion of autoreactive B cells. *J Exp Med* **198**, 1119-26 (2003).
19. Kotzin, J.J. *et al.* The long non-coding RNA Morrbid regulates Bim and short-lived myeloid cell lifespan. *Nature* **537**, 239-243 (2016).
20. Dent, A.L., Shaffer, A.L., Yu, X., Allman, D. & Staudt, L.M. Control of inflammation, cytokine expression, and germinal center formation by BCL-6. *Science* **276**, 589-92 (1997).
21. Hui, C.C. *et al.* Thymic stromal lymphopoietin (TSLP) secretion from human nasal epithelium is a function of TSLP genotype. *Mucosal Immunol* **8**, 993-9 (2015).
22. Corren, J. *et al.* Tezepelumab in Adults with Uncontrolled Asthma. *N Engl J Med* **377**, 936-946 (2017).
23. Parulekar, A.D., Diamant, Z. & Hanania, N.A. Role of T2 inflammation biomarkers in severe asthma. *Curr Opin Pulm Med* **22**, 59-68 (2016).
24. Bonnelykke, K. *et al.* Meta-analysis of genome-wide association studies identifies ten loci influencing allergic sensitization. *Nat Genet* **45**, 902-6 (2013).
25. Ferreira, M.A. *et al.* Genome-wide association analysis identifies 11 risk variants associated with the asthma with hay fever phenotype. *J Allergy Clin Immunol* **133**, 1564-71 (2014).
26. Gudbjartsson, D.F. *et al.* Sequence variants affecting eosinophil numbers associate with asthma and myocardial infarction. *Nat Genet* **41**, 342-7 (2009).
27. Hinds, D.A. *et al.* A genome-wide association meta-analysis of self-reported allergy identifies shared and allergy-specific susceptibility loci. *Nat Genet* **45**, 907-11 (2013).
28. Kottyan, L.C. *et al.* Genome-wide association analysis of eosinophilic esophagitis provides insight into the tissue specificity of this allergic disease. *Nat Genet* **46**, 895-900 (2014).
29. Ramasamy, A. *et al.* A genome-wide meta-analysis of genetic variants associated with allergic rhinitis and grass sensitization and their interaction with birth order. *J Allergy Clin Immunol* **128**, 996-1005 (2011).
30. Rothenberg, M.E. *et al.* Common variants at 5q22 associate with pediatric eosinophilic esophagitis. *Nat Genet* **42**, 289-91 (2010).
31. Sleiman, P.M. *et al.* GWAS identifies four novel eosinophilic esophagitis loci. *Nat Commun* **5**, 5593 (2014).
32. Ochiai, K., Kagami, M., Matsumura, R. & Tomioka, H. IL-5 but not interferon-gamma (IFN-gamma) inhibits eosinophil apoptosis by up-regulation of bcl-2 expression. *Clin Exp Immunol* **107**, 198-204 (1997).
33. Sitkauskiene, B. *et al.* Regulation of bone marrow and airway CD34+ eosinophils by interleukin-5. *Am J Respir Cell Mol Biol* **30**, 367-78 (2004).
34. Ortega, H.G. *et al.* Mepolizumab treatment in patients with severe eosinophilic asthma. *N Engl J Med* **371**, 1198-207 (2014).
35. Ochiai, K. *et al.* Plasmacytic transcription factor Blimp-1 is repressed by Bach2 in B cells. *J Biol Chem* **281**, 38226-34 (2006).
36. Richer, M.J., Lang, M.L. & Butler, N.S. T Cell Fates Zipped Up: How the Bach2 Basic Leucine Zipper Transcriptional Repressor Directs T Cell Differentiation and Function. *J Immunol* **197**, 1009-15 (2016).
37. Vahedi, G. *et al.* Super-enhancers delineate disease-associated regulatory nodes in T cells. *Nature* **520**, 558-62 (2015).

38. Kim, E.H. *et al.* Bach2 regulates homeostasis of Foxp3+ regulatory T cells and protects against fatal lung disease in mice. *J Immunol* **192**, 985-95 (2014).
39. Pai, S.Y., Truitt, M.L. & Ho, I.C. GATA-3 deficiency abrogates the development and maintenance of T helper type 2 cells. *Proc Natl Acad Sci U S A* **101**, 1993-8 (2004).
40. Ting, C.N., Olson, M.C., Barton, K.P. & Leiden, J.M. Transcription factor GATA-3 is required for development of the T-cell lineage. *Nature* **384**, 474-8 (1996).
41. Zheng, W. & Flavell, R.A. The transcription factor GATA-3 is necessary and sufficient for Th2 cytokine gene expression in CD4 T cells. *Cell* **89**, 587-96 (1997).
42. Zhu, J. *et al.* Conditional deletion of Gata3 shows its essential function in T(H)1-T(H)2 responses. *Nat Immunol* **5**, 1157-65 (2004).
43. Ano, S. *et al.* Transcription factors GATA-3 and RORgammat are important for determining the phenotype of allergic airway inflammation in a murine model of asthma. *J Immunol* **190**, 1056-65 (2013).
44. McKenzie, A.N. Type-2 innate lymphoid cells in asthma and allergy. *Ann Am Thorac Soc* **11 Suppl 5**, S263-70 (2014).
45. Kim, P.J. *et al.* GATA-3 regulates the development and function of invariant NKT cells. *J Immunol* **177**, 6650-9 (2006).
