# Supplementary material for: Genome-wide association study of eosinophilic granulomatosis with polyangiitis reveals genomic loci stratified by ANCA status
Source: Nat Commun. 2019 Nov 12;10:5120. doi: 10.1038/s41467-019-12515-9 (PMC6851141; doi:10.1038/s41467-019-12515-9)
Supplement: Supplementary file 8 — Description of Additional Supplementary Files [file 41467_2019_12515_MOESM8_ESM.pdf]

Title: Supplementary Data Item 1

Description: Cross-referencing of EGPA-associated variants with variants in high LD in the NHGRI GWAS Catalog.

Key:

egpa.rsid = rsid of the EGPA associated sentinel variant.
egpa.risk.allele = the risk allele for EGPA of '**egpa.rsid'**
nearest.gene = nearest gene to EGPA sentinel variant
ref_hg19_coordinates = genomic position of the EGPA associated variant (hg19 build)
ref_a1 = the effect allele of the input EGPA-associated variant with respect to the trait in the GWAS Catalog.
ref_a2 = the non-effect allele of the input EGPA-associated variant with respect to the trait in the GWAS Catalog.
gwas_var_rsid = rsid of the trait-associated variant in the GWAS catalog.
gwas_var_hg19_coordinates = genomic position of the trait-associated variant associated variant (hg19 build)
gwas_var_a1 = the effect allele (aligned to the + strand) of the trait-associated variant.
gwas_var_a2 = the non- effect allele (aligned to the + strand) of the trait-associated variant.
proxy = whether the GWAS trait associated variant is an LD proxy for the EGPA SNP. 0= no (i.e. trait-associated variant is exactly the same as the EGPA-associated variant)
r2 = LD (as r2) between EGPA-associated variant and the trait-associated variant based on the phased haplotypes from 1000 Genomes EUR
dprime = LD (as D') between EGPA-associated variant and the trait-associated variant based on the phased haplotypes from 1000 Genomes EUR
trait = trait type
efo = experimental factor ontology term
pmid = the PubMed ID of the study
dataset = dataset from the GWAS study
beta = beta coefficient for genotype from the regression model i.e. estimated effect size of the effect allele in column 'gwas_var_a1'
se = standard error for the genotype coefficient
p = p-value
direction = direction of the allele in column 'gwas_var_a1' on the trait: + = increases; - = decreases.
concordance = directional concordance of the variant on EGPA and the GWAS trait. + indicates same direction; - indicates opposite direction.

Note: where an EGPA sentinel variant from Table 2 does not appear here, this indicates that there is no association with other traits in the GWAS Catalog

Title: Supplementary Data Item 2

Description: cross-referencing of EGPA-associated variants with eQTLs.

Key:

egpa.snp.rsid = rsid of the EGPA associated variant
egpa.risk.allele = the risk allele for EGPA of '**egpa.rsid'**
nearest.gene = nearest gene to EGPA sentinel variant
ref_hg19_coordinates = genomic position of the EGPA associated variant (hg19 build)
ref_a1 = the effect allele of the input EGPA-associated variant with respect to gene expression.
ref_a2 = the non- effect allele of the input EGPA-associated variant with respect to for gene expression.
eqtl.rsid = rsid of the eQTL variant
eqtl_hg19_coordinates = genomic position of the eQTL variant (hg19 build)
eqtl_a1 = the effect allele (aligned to the + strand) for gene expression for the input eQTL variant.
eqtl_a2 = the non-effect allele (aligned to the + strand) for gene expression for the input eQTL variant.
proxy = whether the eQTL variant is an LD proxy for the EGPA SNP. 0= no (i.e. eQTL SNP is exactly the same as the EGPA-associated variant)
r2 = LD (as r2) between EGPA-associated variant and the eQTL variant based on the phased haplotypes from 1000 Genomes

dprime = LD (as D’) between EGPA-associated variant and the eQTL variant based on the phased haplotypes from 1000 Genomes
tissue = tissue in which the eQTL was identified
exp_gene = gene symbol of gene whose expression is associated with the eQTL variant
exp_ensembl = ensemble id of gene whose expression is associated with the eQTL variant
probe = probe id (where gene expression was measured using microarrays)
efo = experimental factor ontology term
study = the eQTL study
pmid = the PubMed ID of the study
dataset = eQTL dataset name
ancestry = study population ancestry
year = year of study
beta = beta coefficient for genotype i.e. estimated effect size of the effect allele
se = standard error for the genotype coefficient
p = p-value
direction = direction of effect allele on gene expression: + = increases; - = decreases.
concordance = directional concordance of the variant on EGPA risk and expression of the gene. + indicates same direction; - indicates opposite direction.

Title: Supplementary Data Item 3

Description: variants associated with traits in NHGRI GWAS Catalog that lie within +/- 1MB of the EGPA-associated variants.
egpa.variant = sentinel EGPA-associated variant
chr = chromosome
egpa.variant.pos = position of sentinel EGPA-associated variant (hg19 build)
trait = trait in GWAS Catalog
trait.assoc.variant.rsid = GWAS trait
gwas.variant.pos = position of GWAS trait-associated variant (hg19 build)
gwas.trait.p.value = p-value for GWAS trait
distance.to.gwas.variant = distance (in bp) from sentinel EGPA-associated variant to GWAS trait-associated variant
pubMedID = PubMed ID
author = first author of study
pubDate = publication date

Title: Supplementary Data Item 4

Description: Mendelian randomisation estimates.
Eosinophil count (EC) was the ‘exposure’ and ‘EGPA’ the outcome.

Method = method used for Mendelian randomisation.
Estimate = MR estimate of the ‘causal’ effect size of a 1 standard deviation increase in EC.
Std Error = standard error.
95% CI = 95% confidence intervals.
P-value = p-value.
